# Modulating Charge Transfer Efficiency of Hematite Photoanode with Hybrid Dual‐Metal–Organic Frameworks for Boosting Photoelectrochemical Water Oxidation

**DOI:** 10.1002/advs.202002563

**Published:** 2020-10-25

**Authors:** Keke Wang, Yang Liu, Kenta Kawashima, Xuetao Yang, Xiang Yin, Faqi Zhan, Min Liu, Xiaoqing Qiu, Wenzhang Li, Charles Buddie Mullins, Jie Li

**Affiliations:** ^1^ School of Chemistry and Chemical Engineering Central South University Changsha 410083 China; ^2^ McKetta Department of Chemical Engineering and Department of Chemistry University of Texas at Austin Austin TX 78712‐0231 USA; ^3^ Institute of Super‐Microstructure and Ultrafast Process in Advanced Materials School of Physics and Electronics Central South University Changsha 410083 China; ^4^ Hunan Provincial Key Laboratory of Efficient and Clean Utilization of Manganese Resources Central South University Changsha 410083 China

**Keywords:** charge transfer efficiency, hematite, hybrid dual‐metal–organic frameworks, water oxidation

## Abstract

The glorious charge transfer efficiency of photoanode is an important factor for efficient photoelectrochemical (PEC) water oxidation. However, it is often limited by slow kinetics of oxygen evolution reaction. Herein, a dual transition metal‐based metal–organic frameworks (MOF) cocatalyst, Fe@Ni–MOF, is introduced into a titanium‐doped hematite (Fe_2_O_3_:Ti) photoanode. The combination of Ni and Fe can optimize the filling of 3d orbitals. Moreover, the introduction of Fe donates electrons to Ni in the MOF structure, thus, suppressing the irreversible (long‐life‐time) oxidation of Ni^2+^ into Ni^3+^. The resulting Fe@Ni–MOF/Fe_2_O_3_:Ti photoanode exhibits ∼threefold enhancement in the photocurrent density at 1.23 V versus the reversible hydrogen electrode. Kinetic analysis of the PEC water oxidation processes indicates that this performance improvement is primarily due to modulating the charge transfer efficiency of hematite photoanode. Further results show that a single transition metal‐based MOF cocatalyst, Ni–MOF, exhibits slow charge transfer in spite of a reduction in surface charge recombination, resulting in a smaller charge transfer efficiency. These findings provide new insights for the development of photoelectrodes decorated with MOFs.

## Graphical abstract

Herein, a hybrid transition metal‐based metal–organic framework (MOF) cocatalyst (Fe@Ni–MOF) is loaded on the surface of a titanium‐doped hematite (Fe_2_O_3_:Ti) photoanode. The introduction of Fe optimizes the filling of 3d orbitals, donating electrons to Ni in the MOF structure. Thus thephotogenerated holes can be transferred to the surface of photoanode more efficiently, which will oxidize water instead of Ni^2+^.

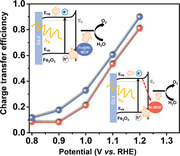

## 1 Introduction

Photoelectrochemical (PEC) water splitting has been deemed as a promising approach to harvest and store solar energy in the form of the chemical energy of hydrogen since the seminal report by Fujishima and Honda.^[^ ^1^, ^2^ ^]^ Generally, a PEC water splitting process is made up of the following three steps: 1) photoexcitation of a semiconductor to generate electron–hole pairs, 2) photoinduced carrier separation and migration, and 3) surface water oxidation and reduction to O_2_ and H_2_ gases by photogenerated holes and electrons, respectively.^[^ ^3^, ^4^ ^]^ To achieve efficient PEC water splitting, it is of supreme importance to develop photoanode materials with a high optical response, as well as effective charge separation and migration, since the water oxidation is a 4‐electron process and represents the bottleneck step for overall water splitting. Over the past several years, many n‐type semiconductor materials, including TiO_2_,^[^ ^5^, ^6^, ^7^ ^]^ WO_3_,^[^ ^8^, ^9^ ^]^ BiVO_4_,^[^ ^10^, ^11^, ^12^, ^13^ ^]^ ZnO,^[^ ^14^, ^15^ ^]^ Fe_2_O_3_,^[^ ^15^, ^16^, ^17^ ^]^ and (oxy)nitrides,^[^ ^18^ ^]^ have been widely investigated as photoanodes for water oxidation. Among all these materials, hematite (*α*‐Fe_2_O_3_) is one of the most promising semiconductors for PEC water oxidation owing to a relatively narrow bandgap (≈2.1 eV), natural abundance, nontoxicity, and chemical stability in basic solution. Theoretically, a 15.3% efficiency for solar‐ to‐hydrogen production and a 12.6 mA cm^−2^ photocurrent density can be achieved at 1.23 V versus the reversible hydrogen electrode (RHE) under AM 1.5 G irradiation.^[^ ^19^ ^]^ However, its performance has been largely constrained by its inherently low conductivity, short lifetimes (less than 10 ps) of photogenerated electrons and holes, and short hole diffusion distance (less than 5 nm).^[^ ^20^ ^]^ Even worse, *α*‐Fe_2_O_3_ suffers from surface charge recombination and sluggish oxygen evolution reaction (OER) kinetics.

Enormous efforts have been devoted to overcome the intrinsic drawbacks and improve the PEC performance of hematite. Researchers have introduced morphology engineering^[^ ^21^ ^]^ to ensure sufficient light absorption and reduce the necessary hole diffuse length, elemental doping^[^ ^22^, ^23^, ^24^ ^]^ (Ti, Sn, P, Si, etc.), constructing heterojunctions^[^ ^25^ ^]^ with other semiconductors, passivating the surface states,^[^ ^26^, ^27^ ^]^ as well as depositing oxygen evolution electrocatalysts (OECs).^[^ ^28^, ^29^, ^30^, ^31^ ^]^ Among these strategies, integrating OECs with *α*‐Fe_2_O_3_ has been confirmed to accelerate the OER kinetics. For example, Zhong and co‐workers reported a *α*‐Fe_2_O_3_ photoanode modified with a CoPi cocatalyst, which has a more negative onset potential and enhanced PEC performance compared to the unmodified *α*‐Fe_2_O_3_ photoanode.^[^ ^32^ ^]^ Rui and co‐workers demonstrated that the MnO_2_ cocatalyst can enhance OER kinetics of a P‐doped Fe_2_O_3_ photoanode with accelerated surface charge separation.^[^ ^33^ ^]^ However, the charge transfer ability of the above‐mentioned cocatalysts is not satisfactory because these cocatalysts inevitably lower the charge separation efficiency due to severe interface recombination and a smaller contact area with the electrolyte.^[^ ^34^ ^]^ A good OECs can not only enhance the PEC water oxidation, but also improve the stability of the photoanodes. Zhang's group developed a low‐cost OEC composed of a phenolic ligand and Ni and Fe ions combined with porous Mo‐doped BiVO_4_ photoanode. Then, a highly stable photocurrent density of ≈5.10 mA cm^−2^ at 1.23 V versus RHE was obtained on the hybrid photoanode.^[^ ^35^ ^]^ Toward this end, integrating NiFe‐based OECs with photoanodes should be beneficial to improve PEC water oxidation performance.^[^ ^36^, ^37^ ^]^

MOFs, which possess distinct properties such as highly porous structures (high surface areas) and designable organic linkers or metal clusters, have attracted considerable attention in electrocatalysis, photocatalysis, and photoelectrocatalysis.^[^ ^38^, ^39^, ^40^ ^]^ In particular, Fe, Co, and Ni‐based MOFs have been designed as an excellent OEC for efficient PEC water splitting.^[^ ^41^, ^42^ ^]^ For instance, a Co–MOF has successfully been fabricated as high‐performance electrocatalysts for the OER due to the Co^II^ active center.^[^ ^43^ ^]^ Indeed, the Ti‐doped Fe_2_O_3_ photoanode modified with a Co‐ZIF‐67 cocatalyst can facilitate water oxidation kinetics.^[^ ^44^ ^]^ Quite recently, dual‐ transition‐metal‐based MOFs (e.g., NiCo, NiFe, CoFe) have attracted attention as OECs.^[^ ^45^, ^46^ ^]^ Compared to single transition metal‐based MOFs, dual‐transition metal‐based MOFs exhibit higher OER performance on account of the coupling effect between different metal species. Previous work has shown that the existence of Fe or Co optimized the filling of e_g_‐orbitals in Ni‐based electrocatalysts,^[^ ^47^ ^]^ thus the NiFe(Co)–MOF demonstrated a superior electrocatalytic activity.^[^ ^46^, ^47^ ^]^ Hence, it is speculated that the dual‐metal‐based MOF OECs coupled with semiconductor materials should provide improvements in PEC water oxidation process.

Herein, we first present a novel dual metal–organic framework as an OER cocatalyst on a titanium‐doped hematite photoanode by a facile solvothermal method [in alkaline media: 1.0 m KOH aqueous solution (pH = 13.4)]. Specifically, Fe@Ni–MOF/Ti‐doped Fe_2_O_3_ was prepared as an OER photoanode. Ti‐doped Fe_2_O_3_ (hereinafter, referred to as Fe_2_O_3_:Ti) was used as a substrate due to its higher charge transfer ability in the bulk compared to undoped Fe_2_O_3_.^[^ ^48^, ^49^ ^]^ The Fe@Ni–MOF was chosen as an OEC owing to its earth abundance, environmentally friendliness, and high corrosion resistance.^[^ ^50^ ^]^ Furthermore, the combination of Ni and Fe can optimize the 3d orbital filling, thus benefiting the OER activity. Simultaneously, the molar ratio of Fe:Ni can be controlled by tuning the concentration of Fe and Ni precursor solution during the solvothermal process. Our champion Fe@Ni–MOF/Fe_2_O_3_:Ti photoanode was ultimately able to give rise to better performance for PEC water oxidation. Additionally, we further investigated the surface charge carrier dynamics of the unmodified Fe_2_O_3_:Ti, Ni–MOF, and Fe@Ni–MOF decorated Fe_2_O_3_:Ti photoanodes. The analytical results indicated that the Fe@Ni–MOF/Fe_2_O_3_:Ti photoanode could accelerate the surface charge transfer, which makes better use of the surface‐reaching holes for water oxidation. For the Ni–MOF/Fe_2_O_3_:Ti photoanode, contrastingly, the slow charge transfer rate resulted in a loss of surface‐reaching holes, which is unfavorable for further improvements in PEC performance. Our analysis indicates that an optimal PEC performance of Fe_2_O_3_:Ti is acquired from a fine balance between surface charge transfer and recombination rate.

## 2 Results and Discussion

### 2.1 Morphological and Compositional Characterization of Fe@Ni–MOF/Fe_2_O_3_:Ti Photoanode

**Figure 1:**
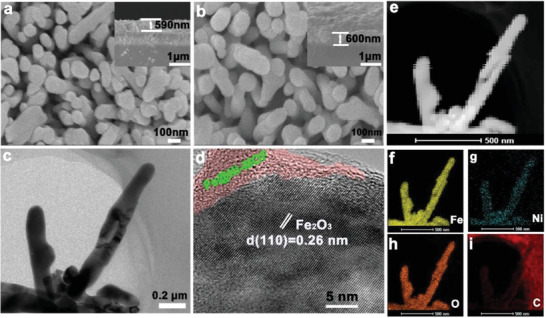
Top‐view and cross‐sectional (inset) SEM images of a) Fe_2_O_3_:Ti and b) Fe@Ni–MOF(Fe:Ni = 1:10)/Fe_2_O_3_:Ti nanorod arrays, c) TEM, d) HR‐TEM, e) HAADF‐STEM, and f–i) the corresponding HR‐TEM elemental mappings images of Fe@Ni–MOF/Fe_2_O_3_:Ti composites.

In this work, the Fe@Ni–MOF/Fe_2_O_3_:Ti photoanodes were synthesized via the direct solvothermal growth of Fe@Ni–MOF on *α*‐Fe_2_O_3_:Ti nanorod surface with different molar ratios of Fe:Ni. The preparation process of the Fe@Ni–MOF/Fe_2_O_3_:Ti photoanode is depicted in Figure S1 (Supporting Information). The morphology and microstructure of the Fe_2_O_3_:Ti and representative Fe@Ni–MOF (Fe:Ni = 1:10)/Fe_2_O_3_:Ti samples were observed by using a field‐emission scanning electron microscope and transmission electron microscope (TEM). As presented in **Figure** **1**a, the Fe_2_O_3_:Ti retains a 1D nanorod structure with an average diameter of 54.2 ± 11.3 nm (Figure S2, Supporting Information). The length of Fe_2_O_3_:Ti nanorods is also observed according to the cross‐sectional view (inset of Figure 1a, ≈590 nm). After modifying with Fe@Ni–MOF, the surfaces of the Fe_2_O_3_:Ti nanorods become rough (Figure 1b). The corresponding average diameter increases to 64.6 ± 10.6 nm (Figure S2, Supporting Information), while the length of the Fe@Ni–MOF/Fe_2_O_3_:Ti nanorods (inset of Figure 1b, ≈600 nm) is shown to be very close to that of Fe_2_O_3_:Ti nanorods. In addition, the morphology of Fe@Ni–MOF/Fe_2_O_3_:Ti nanocomposites with different molar ratios of Fe:Ni was also measured. As shown in Figure S3a–f (Supporting Information), the outermost surfaces of the Fe_2_O_3_:Ti arrays are covered with a thick layer of Fe@Ni–MOF. The TEM and high‐resolution TEM (HRTEM) were conducted to further confirm the presence of Fe@Ni–MOF. The 1D rod‐like morphology of the as‐synthesized Fe_2_O_3_:Ti is affirmed and displayed in Figure S4 (Supporting Information). In the Fe@Ni–MOF/Fe_2_O_3_:Ti composite, a conformal shell ≈10 nm covering the Fe_2_O_3_:Ti is found (Figure 1c,d). Additionally, the lattice fringe spacing of 0.26 nm from the HRTEM image belongs to the (110) plane of hematite. Furthermore, the HR‐TEM elemental mapping analysis demonstrates that both Ni and C are in the exterior outline of the nanorod while Fe and O are homogeneously distributed throughout the array (Figure 1e–i). Finally, the energy‐dispersive X‐ray spectroscopy characterization of Fe@Ni–MOF powder also indicates that Fe, Ni, Co, and O elements exist in the Fe@Ni–MOF/Fe_2_O_3_:Ti composite, and the atomic percent of Ni is ≈27 times higher than that of Fe (Figure S5, Supporting Information).

### 2.2 Structure and Surface Chemistry Characterization of Fe@Ni–MOF/Fe_2_O_3_:Ti Photoanode

**Figure 2:**
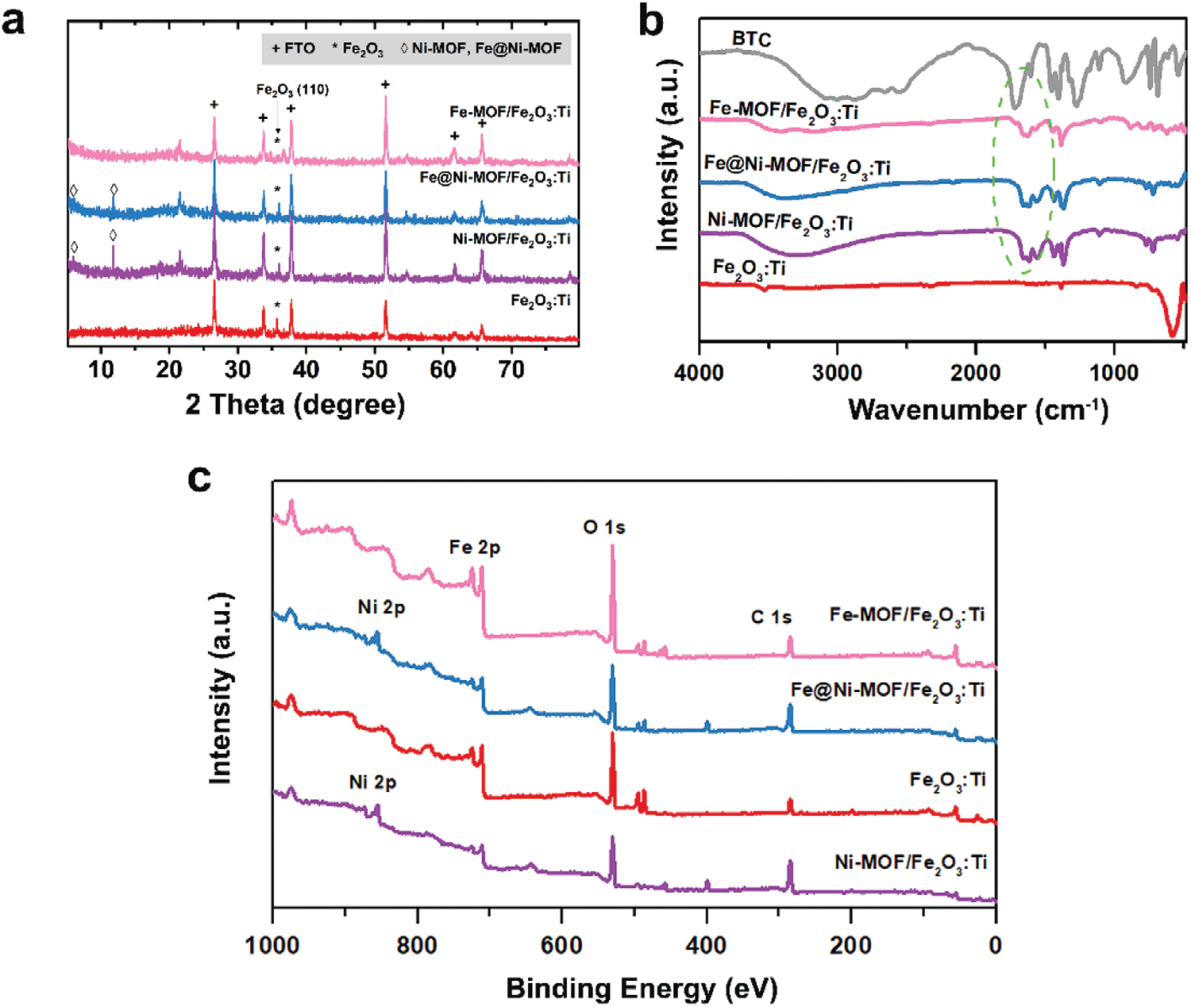
a) XRD patterns, b) FT‐IR spectra, and c) XPS spectra of Fe_2_O_3_:Ti, Ni–MOF/Fe_2_O_3_:Ti, Fe@Ni–MOF(Fe:Ni = 1:10)/Fe_2_O_3_:Ti, and Fe–MOF/Fe_2_O_3_:Ti photoanodes.

The resultant crystalline and chemical structures composition of all the as‐prepared samples were then confirmed by X‐ray diffraction (XRD) and Fourier transform infrared (FT‐IR) spectroscopy. As seen in **Figure** **2**a, in addition to the peaks from the fluorine‐doped tin oxide (FTO) substrate, there is a clear diffraction peak at 33.6°, which can be well assignable to *α*‐phase Fe_2_O_3_ (JCPDS no. 33‐0664) with preferential oriented growth of the (110) plane.^[^ ^51^ ^]^ It is worth noting that Fe@Ni–MOF/Fe_2_O_3_:Ti composite and Ni–MOF/Fe_2_O_3_:Ti show similar XRD peaks (at around 5.7, and 11.6°), which correspond to Fe@Ni–MOF (Figure S6, Supporting Information). In contrast, no obvious diffraction peaks can be found for pristine Fe–MOF, implying its amorphous nature.^[^ ^50^ ^]^ The FT‐IR spectra are also provided in Figure 2b, the as‐prepared Fe_2_O_3_:Ti photoanodes possess a peak at around 500 cm^−1^, which could be attributed to the vibrational peak of Fe–O. After the growth of MOFs, the carboxylic group of the trimesic acid stretching vibration at around 1610 cm^−1^ is observed.^[^ ^52^ ^]^ The results demonstrate that Fe@Ni–MOFs were successfully formed on the surface of the Fe_2_O_3_:Ti nanorods.

To provide proof for the formation of Fe@Ni–MOF, X‐ray photoelectron spectroscopy (XPS) analysis was also employed. The powder XPS results of the Fe@Ni–MOFs are available in Figure S7 (Supporting Information). The high‐resolution Ni 2p spectrum demonstrates the presence of Ni^2+^ (Ni 2p_3/2_ ≈855.7 eV, Ni 2p_3/1_ ≈873.3 eV), while the Fe 2p doublet (Fe 2p_3/2_ ≈711.7 eV, Fe 2p_1/2_ ≈724.0 eV) can also be found in Figure S7 (Supporting Information), which confirm the presence of Fe@Ni–MOF. As shown in Figure 2c, the predominant peaks of Fe 2p, Ni 2p, Ti 2p, O 1s, and C 1s were observed in the survey spectrum of Fe@Ni–MOF/Fe_2_O_3_:Ti, implying the existence of both Fe_2_O_3_:Ti and Fe@Ni–MOF in the as‐fabricated photoanodes.

### 2.3 PEC Water Oxidation Performance

**Figure 3:**
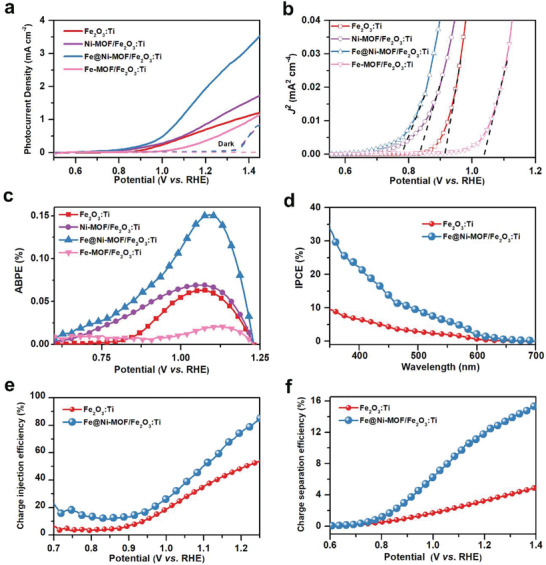
a) *J–V* curves, b) Bluter plots, and c) Applied bias photon‐to‐current conversion efficiency (ABPE) for Fe_2_O_3_:Ti, Ni–MOF/Fe_2_O_3_:Ti, Fe@Ni–MOF(Fe:Ni = 1:10)/Fe_2_O_3_:Ti, and Fe–MOF/Fe_2_O_3_:Ti photoanodes under simulated AM 1.5 G sunlight irradiation, d) Incident photon‐to‐current efficiency (IPCE) spectra, e) charge injection efficiencies, and f) charge separation efficiency of the Fe_2_O_3_:Ti and Fe@Ni–MOF/Fe_2_O_3_:Ti photoanodes.

**Figure** **3**a shows the photocurrent density–potential (*J–V*) plots of the as‐fabricated photoanodes. The unmodified Fe_2_O_3_:Ti showed a photocurrent density of 0.76 ± 0.02 mA cm^−2^ at 1.23 V versus RHE. When the Ni–MOF was deposited on the Fe_2_O_3_:Ti nanorods, the sample exhibits a slight negative shift and increases in the onset‐potential and photocurrent density, respectively. Then, the higher photocurrent density of the Ni–MOF/Fe_2_O_3_:Ti photoanode is recorded at a relatively high potential in comparison to the Fe_2_O_3_:Ti photoanode, which is due to the high electrochemical OER activity of the Ni‐based electrocatalyst.^[^ ^53^ ^]^ Upon Fe incorporation into the Ni–MOF on the Fe_2_O_3_:Ti photoanode, a further increase of photocurrent density is observed over the entire applied potential region. It may be attributed to the improved charge transfer efficiency due to Fe incorporation.^[^ ^54^, ^55^ ^]^ Nevertheless, it is worth mentioning that the Fe–MOF/Fe_2_O_3_:Ti delivers the lowest photocurrent density out of all the measured samples, which can be ascribed to the poor oxygen evolution activity of Fe–MOF.^[^ ^56^ ^]^ Moreover, to obtain optimal PEC performance, we control the amount of Fe incorporated into Ni–MOF, and the Fe_2_O_3_:Ti with the MOF samples were fabricated with different Fe:Ni molar ratios (1:1, 1:5, 1:10, and 1:15). As shown in Figure S8 (Supporting Information), both enhanced photocurrent densities and reduced onset potentials are achieved with the relatively large Fe incorporation amounts (Fe:Ni = 1:1, 1:5, and 1:10). However, the photocurrent density begins to decrease with reduced contents of Fe (Fe:Ni = 1:15). The optimal PEC performance can be obtained at the Fe:Ni molar ratio of 1:10 (Figure 3b). Moreover, the Fe@Ni–MOF(1:10)/Fe_2_O_3_:Ti photoanode shows the highest photocurrent density of 2.3 ± 0.06 mA cm^−2^ at the potential of 1.23 V versus RHE, which is about threefold higher than that of the Fe_2_O_3_:Ti photoanode.

The applied bias photon‐to‐current efficiency (ABPE) was also obtained by calculating the data from the as‐measured polarization curves. As shown in Figure 3c, the Fe@Ni–MOF/Fe_2_O_3_:Ti photoanode achieves the maximum ABPE values of 0.15%, which is ≈2.5 times higher than that of the unmodified Fe_2_O_3_:Ti. To further study the impact of the Fe@Ni–MOF shell on the PEC performance, incident photo‐to‐electron conversion efficiency (IPCE) measurements were conducted to evaluate the external quantum efficiency. Compared with the bare Fe_2_O_3_:Ti photoanode, the Fe@Ni–MOF/Fe_2_O_3_:Ti photoanode records higher IPCE values over the measured wavelength range of 350–610 nm (Figure 3d). In particular, the IPCE value of the Fe@Ni–MOF/Fe_2_O_3_:Ti photoanode is 34.2% at 350 nm, which is ≈3.5 fold of the bare Fe_2_O_3_:Ti photoanode (9.6%). To our best knowledge, the as‐obtained IPCE value is closely relevant to the theoretical light harvesting capability and charge separation as well as injection efficiencies of Fe_2_O_3_. Based on the results from the UV–vis absorption spectra, there is no significant difference among the samples, indicating that light harvesting property is not the factor for the enhanced PEC performance (Figure S9a,b, Supporting Information).

The charge separation and injection efficiencies were also estimeted by a hole‐scavenger‐assisted PEC measurement. As shown in Figure 3e, the highest charge injection efficiency (*η*_inj_) can be observed for the Fe@Ni–MOF/Fe_2_O_3_:Ti photoanode, implying that the surface charge recombination might be significantly suppressed. Furthermore, the Fe@Ni–MOF/Fe_2_O_3_:Ti photoanode exhibited superior charge separation efficiency (*η*_sep_) over the entire range of potentials compared with the unmodified Fe_2_O_3_:Ti photoanode. These results indicate that the Fe@Ni–MOF shell could promote charge transfer capacity in the porous structure of the MOF and reduce surface charge recombination. The light response measurement was also applied to evaluate the repeatability and photosensitivity of the as‐prapared photoanodes. As shown in Figure S10 (Supporting Information), the as‐prepared photoanodes displayed the sensitive light responses to the illumination during the several light on‐off cycles, repeatedly. As expected, the corresponding photocurrent values followed a similar trend in the *J–V* curves. Importantly, the Fe@Ni–MOF/Fe_2_O_3_:Ti photoanode could successfully manifest excellent PEC performance for the OER, which is comparable or even superior to the majority of the reported Fe_2_O_3_ photoanodes modified with cocatalysts (Table S1, Supporting Information).

Photostability is also an important factor for evaluating PEC performance toward water oxidation. As seen in Figure S11a (Supporting Information), the Fe@Ni–MOF/Fe_2_O_3_:Ti photoanode exhibits a negligible loss in photocurrent during the 2 h test, suggesting its outstanding photostability. Furthermore, the produced H_2_ and O_2_ gases from the Fe–MOF/Fe_2_O_3_:Ti photoanode are collected and quantified. The as‐measured curves of the evolved gases are shown in Figure S11b (Supporting Information). The amount of evolved O_2_ and H_2_ linearly increases with the increase of illumination time and the O_2_:H_2_ molar ratio is around 1:2, which is close to the theoretical value. The discrepancy with the theoretical result can stem from the inevitable O_2_ dissolution in the electrolyte inevitably. The faraday efficiency is determined to be ≈92% when the system achieves a steady state. Accordingly, the photostalibity test results reveal that the Fe@Ni–MOF cocatalyst can stably utilize the photogenerated holes to produce O_2_ gas in our system.

To gain further insights into the resultant enhancement of the PEC OER performance, the possible factors affecting the catalytic activity were explored thoroughly. The elecrochemically active surface areas (ECSAs) of the as‐prepared samples were estimated according to the double‐layer capacitance (*C*_dl_), which is based on cyclic voltammetry (CV) measurement in the non‐Faradaic potential region. Specifically, a series of CV tests were carried out at different scan rates (20, 40, 60, 80, and 100 mV s^−1^) within 0.6–0.8 V versus RHE (Figure S12a–d, Supporting Information). The calculated ECSAs of the Fe_2_O_3_:Ti, Ni–MOF/Fe_2_O_3_:Ti, Fe@Ni–MOF/Fe_2_O_3_:Ti, and Fe–MOF/Fe_2_O_3_:Ti samples are 1.08 ± 0.012, 2.78± 0.045, 3.25 ± 0.063, and 1.99 ± 0.010 cm^2^, respectively. As shown in Figure S13 (Supporting Information), the renormalized photocurrent density (based on ECSA) of the Ni–MOF/Fe_2_O_3_:Ti photoanode is lower than the Fe_2_O_3_:Ti photoanode. On the contrary, the Fe@Ni–MOF/Fe_2_O_3_:Ti photoanode shows a higher photocurrent density (based on ECSA) than the Fe_2_O_3_:Ti photoanode, implying that the Fe@Ni–MOF cocatalyst has quite high intrinsic OER activity and the Fe@Ni–MOF cocatalyst loading is conducive to accelerate the charge transfer rate and reduce charge recombination in Fe_2_O_3_:Ti.

### 2.4 Kinetic Characterization of Charge Transfer

**Figure 4:**
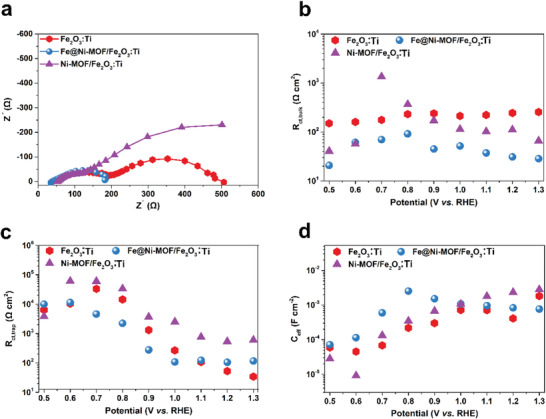
a) PEIS Nyquist plots (under illumination at 1.0 V vs RHE) and b–d) fitting results from PEIS measurements at different applied potentials: b) *R*_ct,bulk_, c) *R*_ct,trap_, and d) *C*_eff_.

Generally, in a simplified PEC system, it is perceived that there are two competitive processes near a semiconductor electrode surface, namely charge transfer and surface recombination. Hence, the corresponding PEC impedance spectra (PEIS) consists of two semicircles located at high frequency (HF) and low frequency (LF), respectively. As presented in **Figure** **4**a, the semicircle situated at HF represents the charge transfer resistance in the hematite photoanode, and the other one at LF is in connection with the charge transfer resistance at the photoanode/electrolyte interface. It can be observed that the diameter of the semicircle (at LF) of the Fe@Ni–MOF/Fe_2_O_3_:Ti photoanode is much smaller relative to the Ni–MOF/Fe_2_O_3_:Ti and Fe_2_O_3_:Ti photoanodes, meaning that the Fe@Ni–MOF cocatalyst can greatly facilitate the charge transfer at the photoanode/electrolyte interface and thus the electron–hole recombination may be dramatically inhibited.

Here, the proposed equivalent circuit is shown in Figure S14 (Supporting Information). In Figure 4b, *R*_ct,bulk_ (charge transfer resistance in the bulk hematite photoanode) values of the Fe@Ni–MOF/Fe_2_O_3_:Ti and Ni–MOF/Fe_2_O_3_:Ti photoanodes are lower in comparison to the Fe_2_O_3_:Ti photoanode, manifesting that these loaded MOF‐based cocatalysts might accelerate the charge transfer from Fe_2_O_3_:Ti to the MOF. Meanwhile, a lower *R*_ct,trap_ value for the Fe@Ni–MOF/Fe_2_O_3_:Ti photoanode is obtained from 0.7 to 1.05 V versus RHE (Figure 4c), which can be attributed to the suppression of the hole pinning on the electrode surface by the Fe@Ni–MOF cocatalyst.^[^ ^57^ ^]^ Moving toward the higher potential, *R*_ct,trap_ (charge transfer resistance across the electrolyte/hematite interface) values for both the Fe_2_O_3_:Ti and Fe@Ni–MOF/Fe_2_O_3_:Ti photoanodes are almost the same because charge transfer to the electrolyte might not be a limiting process. However, the Ni–MOF/Fe_2_O_3_:Ti photoanode has a larger *R*_ct,trap_ value, suggesting a slower charge transfer rate within the Ni–MOF material. In the case of the Ni–MOF, a large portion of the photogenerated holes might recombine instead of facilitate OER. Figure 4d displays the tendency of effective capacitances (*C*_eff_) at the photoanode/electrolyte interfaces for the Fe_2_O_3_:Ti, Ni–MOF/Fe_2_O_3_:Ti, and Fe@Ni–MOF/Fe_2_O_3_:Ti photoanodes. As for the Fe_2_O_3_:Ti and Ni–MOF/Fe_2_O_3_:Ti photoanodes, their *C*_eff_ values are increased at around 0.70–1.05 V versus RHE, which occur in conjunction with their *R*_ct,trap_‐*V* value decreases. This phenomenon can be attributed to the high ionic permeability of the Fe@Ni–MOF shell.^[^ ^53^ ^]^ The increased *C*_eff_ value for the Ni–MOF/Fe_2_O_3_:Ti photoanode may be interpreted as hole storage within the Ni–MOF. Besides, the Fe@Ni–MOF/Fe_2_O_3_:Ti photoanode shows the largest cathodic shift, which is coincidently correlated with the onset potential (Figure 3b).

To understand the behavior of capturing holes by the Fe@Ni–MOF shell, the Bode plots of the unmodified Fe_2_O_3_:Ti and Fe@Ni–MOF/Fe_2_O_3_:Ti photoanodes were obtained at different potentials (Figure S15, Supporting Information). Below 1.0 V versus RHE, both electrodes exhibit higher phase values at LF, indicating charge transfer is limited by the photoanode/electrolyte interface during PEC water oxidation. Additionally, the peaks for the Fe@Ni–MOF/Fe_2_O_3_:Ti photoanode are located at slightly lower frequencies than those for the Fe_2_O_3_:Ti photoanode, implying the holes are effectively captured by the Fe@Ni–MOF shell. Above 1.0 V versus RHE, the peak intensity of the Fe_2_O_3_:Ti at LF is reduced and located at a higher frequency than that of the Fe@Ni–MOF/Fe_2_O_3_:Ti, implying that the limiting process is the charger transfer in the Fe_2_O_3_:Ti bulk rather than the charge transfer at the photoanode/electrolyte interface.

As mentioned above, there are two processes: charge transfer and surface recombination occurring at the surfaces of the Fe_2_O_3_:Ti‐based photoanodes during the OER. The corresponding rate constants have been described as the charge‐transfer rate constant (*K*_ct_) and surface recombination rate constant (*K*_rec_) according to the reported phenomenological model.^[^ ^58^, ^59^ ^]^ *K*_ct_ can be obtained by Equation (1), supposing the capacitance of the Helmholtz layer is much higher than the space charge capacitance of the photoanode^[^ ^60^ ^]^

(1) $$\begin{array}{c} K_{\mathrm{ct}}=1/R_{\mathrm{LF}}C_{\mathrm{LF}} \end{array}$$

where *R*_LF_ and *C*_LF_ are defined as the resistance and capacitance at low frequency, respectively. They can be calculated according to the following Equations (2) and (3)

(2) $$\begin{array}{c} R_{\mathrm{LF}}=\frac{K_{B}T}{q^{2}J_{h}}\;\left(\frac{K_{\mathrm{ct}}+K_{\mathrm{rec}}}{K_{\mathrm{ct}}}\right) \end{array}$$

(3) $$\begin{array}{c} C_{\mathrm{LF}}=\frac{q^{2}J_{h}}{K_{B}T}\;\left(\frac{1}{K_{\mathrm{ct}}+K_{\mathrm{rec}}}\right) \end{array}$$

in which *K*_B_ represents the Boltzmann constant, *T* expresses the temperature, *q* is the elementary charge, and *J*_h_ denotes the flux of holes which reach the photoanode/electrolyte interface (i.e., the measured current density).

Meanwhile, *K*_rec_ can also be calculated by the following Equation (4)

(4) $$\begin{array}{c} K_{\mathrm{rec}}=\frac{R_{\mathrm{LF}}}{R_{\mathrm{HF}}}\;K_{\mathrm{ct}} \end{array}$$

**Figure 5:**
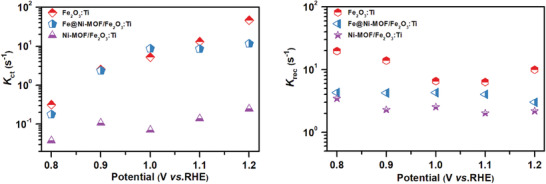
a) Charge transfer (*K*_ct_) and b) charge recombination (*K*_rec_) rates for Fe_2_O_3_:Ti, Fe@Ni–MOF(Fe:Ni = 1:10)/Fe_2_O_3_:Ti, and Ni–MOF/Fe_2_O_3_:Ti photoanodes at different applied potentials.

Where *R*_HF_ is the resistance of high frequency. As shown in **Figure** **5**a. *K*_ct_ is independent of the potential, which is consistent with a previous report.^[^ ^61^ ^]^ It is important to note that *K*_ct_ of the Ni–MOF/Fe_2_O_3_:Ti is smaller than that of the Fe_2_O_3_:Ti photoanode. One promissing explanation is that the Ni–MOF cocatalyst cannot capture the available surface‐reaching holes, resulting in a turnover frequency decrease and the hole retention time prolongation.^[^ ^62^ ^]^ This would further increase the opportunity for the interfacial recombination of the photogenerated charges. To evaluate the device performance, the charge transfer efficiency (*Φ*_ct_) can be acquired through Equation (5)

(5) $$\begin{array}{c} \Phi_{\mathrm{ct}}=\frac{K_{\mathrm{ct}}}{K_{\mathrm{rec}}+K_{\mathrm{ct}}}\; \end{array}$$

Even though a decrease in *K*_rec_ (Figure 5b) is also found for the Ni–MOF/Fe_2_O_3_:Ti photoanode, its *Φ*_ct_ as well as PEC performance are still restricted by the slow charge transfer rate. Furthermore, Fe@Ni–MOF/Fe_2_O_3_:Ti exhibits notably decreased *K*_rec_ values with a slight change in the *K*_ct_ values compared to the Fe_2_O_3_:Ti photoanode. Thus, a much higher *Φ*_ct_ is achieved after incorporating Fe into the Ni–MOF (Figure S16, Supporting Information).

**Figure 6:**
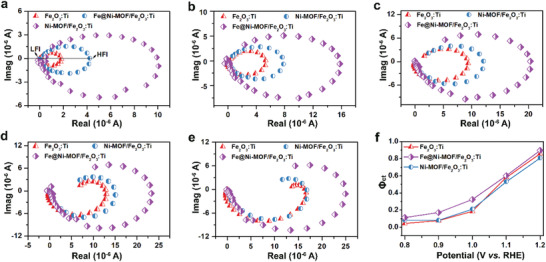
IMPS spectra for Fe_2_O_3_:Ti (red), Ni–MOF/Fe_2_O_3_:Ti (purple), and Fe@Ni–MOF(Fe:Ni = 1:10)/Fe_2_O_3_:Ti photoanodes (blue) at the potentials of a) 0.8, b) 0.9, c) 1.0, d) 1.1, and e) 1.2 V versus RHE. f) Calculated charge transfer efficiency for different photoanodes.

Intensity modulated photocurrent spectroscopy (IMPS) measurements were also conducted to support the PEIS results. As shown in **Figure** **6**a–e, there are two semicircles in the IMPS spectra corresponding to HF and LF intersects with the real axis (HFI and LFI), respectively. In general, HFI represents the flux of holes to the surface, while LFI is related to the transfered charge at the photoanode/electrolyte interface.^[^ ^63^, ^64^ ^]^ Concretely speaking, at 0.8–1.2 V versus RHE, the Fe@Ni–MOF/Fe_2_O_3_:Ti photoanode shows a larger HFI than the Ni–MOF/Fe_2_O_3_:Ti and Fe_2_O_3_:Ti photoanodes, suggesting a higher hole flux at the Fe@Ni–MOF/Fe_2_O_3_:Ti surface. This phenomenon illustrates that the band bending in the space charge region is more noticeable after the Fe@Ni–MOF deposition on account of faster charge transfer from the Fe_2_O_3_:Ti to the Fe@Ni–MOF shell. The LFI value of the Fe@Ni–MOF/Fe_2_O_3_:Ti photoanode is much smaller than those of the Ni–MOF/Fe_2_O_3_:Ti and unmodified Fe_2_O_3_:Ti photoanodes, implying that the incorporation of Fe into Ni–MOF may facilitate charge transfer and reduce interface charge recombination. As we know, the ratio of LFI and HFI signifies the proportion of holes reaching the surface to holes injected into the electrolyte, which is called charge transfer efficiency. The corresponding formula is shown as follows

(6) $$\begin{array}{c} \Phi_{\mathrm{ct}}=\frac{K_{\mathrm{ct}}}{K_{\mathrm{ct}}+K_{\mathrm{rec}}}\;=\frac{\mathrm{LFI}}{\mathrm{HFI}}\; \end{array}$$

As shown in Figure 6f, the Fe@Ni–MOF/Fe_2_O_3_:Ti photoanode shows the highest charge transfer efficiency among the as‐prepared samples. It is a remarkable fact that the charge transfer efficiency of the Ni–MOF/Fe_2_O_3_:Ti photoanode is comparable to that of the Fe_2_O_3_:Ti photoanode.To further confirm this interpretation, a transient photocurrent study was also conducted (Figure S17, Supporting Information). At low potentials, the MOF‐modified Fe_2_O_3_:Ti photoandes exhibit slower photocurrent decay curves, suggesting that the holes reaching the surface are trapped by the MOFs. At high potentials, the spike of the Fe_2_O_3_:Ti photoanode becomes almost unapparent. At all the potentials, the Ni–MOF/Fe_2_O_3_:Ti photoanode presented a faster photocurrent decay. This suggests that the holes accumulated on the Ni–MOF are recombined immediately. After introducing Fe into the Ni–MOF, the accumulation of photogenerated holes is decreased due to the faster charge transfer so that the recombination is relieved.

### 2.5 Verification of the Interaction between Fe and Ni

**Figure 7:**
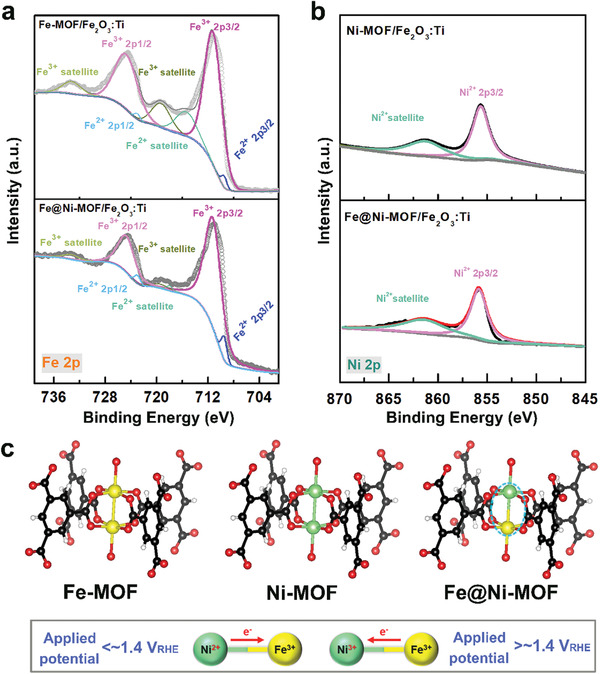
High‐Resolution XPS spectra of a) Fe 2p in Fe–MOF/Fe_2_O_3_:Ti and Fe@Ni–MOF/Fe_2_O_3_:Ti, b) Ni 2p in Ni–MOF/Fe_2_O_3_:Ti and Fe@Ni–MOF/Fe_2_O_3_:Ti. c) Interaction of Ni and Fe during the water oxidation process.

**Figure 8:**
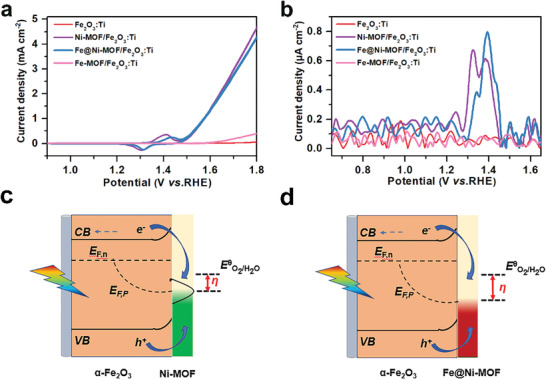
a) Dc cyclic voltammograms (dark) and b) FT ac voltammograms for unmodified and MOF‐modified Fe_2_O_3_:Ti photoanodes. Simplified models of the basic PEC OER processes in c) Ni–MOF/Fe_2_O_3_:Ti and d) Fe@Ni–MOF/Fe_2_O_3_:Ti photoanodes.

To further certify the interaction of both Fe and Ni in the Fe@Ni–MOF, XPS results are shown in Figure 8a,b. Here, the partial electron transfer from Ni to Fe can be observed, which is supported by a negative shift of Fe^3+^ peaks (710.9 and 724.4 eV, **Figure** **7**a) and positive shift of Ni^2+^ peaks (855.9 and 861.6 eV, Figure 7b) for Fe@Ni–MOF/Fe_2_O_3_:Ti compared to Fe–MOF/Fe_2_O_3_:Ti (711.3 and 724.7 eV, Figure 7a), and Ni–MOF/Fe_2_O_3_:Ti (855.6 and 861.3 eV, Figure 7b),respectively.^[^ ^47^ ^]^ Generally, the electronegativity of Ni (*χ*_Ni_ = 1.91) is slightly larger than that of Fe (*χ*_Fe_ = 1.83). Thus, it is expected that electrons may transfer from Fe to Ni. However, Ni and Fe in the MOF mainly exhibit the oxidation states 2+ and 3+, respectively. Fe^3+^ has a higher electron deficiency than Ni^2+^, which may let electrons transfer from Ni^2+^ to Fe^3+^ (Figure 7c). The MOF structures in Figure 7c were illustrated by using *VESTA 3* software.^[^ ^65^ ^]^ Moreover, introduction of Fe can increase the valence of Ni according to a previous report about Ni–Fe oxide.^[^ ^55^ ^]^ The Ni with a higher valence state may contribute a stronger charge transfer process between OH^−^ and the Fe–Ni–MOF. Hence, the interaction of both Ni and Fe may be able to optimize the d‐orbital filling and charge transfer ability, which can improve the catalytic activity.^[^ ^46^, ^66^ ^]^ Additionally, the small Fe^2+^ peaks can also be found in the Fe–MOF and Fe@Ni–MOF (Figure 7a), which matches a previous report.^[^ ^67^ ^]^

In Figure S18 (Supporting Information), a schematic illustration of the proposed OER mechanism in the transition metal (*M*)‐MOF is presented. Herein, the corresponding OER steps in alkaline solution are described as below^[^ ^68^ ^]^

(7) $$\begin{array}{c} {}^{*}+OH^{-}+h^{+}\to {\mathrm{OH}}^{*} \end{array}$$

(8) $$\begin{array}{c} {\mathrm{OH}}^{*}+OH^{-}+h^{+}\to O^{*}+H_{2}O \end{array}$$

(9) $$\begin{array}{c} {}^{*}O+OH^{-}+h^{+}\to {\mathrm{OOH}}^{*} \end{array}$$

(10) $$\begin{array}{c} {\mathrm{OOH}}^{*}+OH^{-}+h^{+}\to O_{2}+H_{2}O{+}^{*} \end{array}$$

where * represents a *M* active site. According to the above reaction pathway, the available holes are conductive to OER. Thus, the interaction between Ni and Fe (Figure 7c), which is in favor of transforming photogenerated holes to the reaction active sites, promoting the water oxidation reaction.

To get a better understanding of the influence of Fe@Ni–MOF on PEC water oxidation, the direct current (dc) cyclic voltammograms and Fourier transformed (FT) alternating current (ac) voltammograms are provided in **Figure** **8**. The dc cyclic voltammogram (Figure 8a) displays redox peaks corresponding to the transition between Ni^2+^ and Ni^3+^. When Fe is incorporated into Ni–MOF, obvious positive shift of the peak observed, which is close to the water oxidation potential. This phenomenon can be interpreted from the Fe incorporation which may alter the redox properties of Ni. The similar phenomenon was reported in the Fe modified Ni‐based OEC.^[^ ^69^ ^]^ FT ac voltammograms (Figure 8b), which are helpful in probing the electron transfer aspects associated with electrocatalytic reactions, also show a positive shift of the Ni^2+^/Ni^3+^ redox peak for Fe@Ni–MOF/Fe_2_O_3_:Ti compared to Ni–MOF/Fe_2_O_3_:Ti. The Ni^2+^/Ni^3+^ oxidation is not deemed as being directly involved in the PEC OER. Instead, the transformation of Ni^2+^ to Ni^3+^ will consume the photogenerated holes.

As shown in Figure 8c, photogenerated holes that arrive at the surface of the Fe_2_O_3_:Ti photoanode participate in the fast oxidation of Ni^2+^/Ni^3+^ in the Ni–MOF cocatalyst, and then the further oxidation to Ni^4+^ occurs as a rate limiting step. Thus, the Ni–MOF cocatalyst might accumulate holes rather than release holes to proceed with the OER.^[^ ^57^ ^]^ This is the reason for the fact that the charge transfer efficiency of the Ni–MOF/Fe_2_O_3_:Ti photoanode is comparable to that of the Fe_2_O_3_:Ti photoanode (Figure 6f). Notably, this low OER performance of the Ni–MOF/Fe_2_O_3_:Ti photoanode is associated with the holes being stored and then participating in the redox cycle of Ni^2+^/Ni^3+^, resulting in its fast interfacial recombination. The Fe incorporation can shift the Ni^2+^/Ni^3+^ potential toward higher potential and reduce the rate of the interface charge recombination, thus enhancing the charge transfer efficiency. On the other hand, for the Fe@Ni–MOF/Fe_2_O_3_:Ti, the introduced Fe can donate electrons to Ni in the MOF structure due to a high electronegativity of Ni under the oxidized bias > ∼1.4 V (Figure 7c). According to the recent in situ study,^[^ ^30^ ^]^ Fe species in the Ni–Fe oxide electrocatalysts maintain the oxidation state of +3 even at the high‐applied potential. Hence, the Fe in the MOF may also consistently remain in the +3 states due to the electron delocalization. At a high applied potential, this Fe^3+^ in the Fe^3+^@Ni^2+^–MOF may suppress the irreversible (long‐life‐time) oxidation of Ni^2+^ into Ni^3+^ by donating its electrons. Herein, formation of Ni^3+^ probably traps the photogenerated holes. Hence, the Fe@Ni–MOF/Fe_2_O_3_:Ti shows slightly smaller Ni^2+^/Ni^3+^ redox peak areas, in which the peak area of Ni^3+^ reduction seems as large as that of Ni^2+^ oxidation, compared with the Ni–MOF/Fe_2_O_3_:Ti. Note that in the Ni–MOF, the peak area for Ni^3+^ reduction is visually smaller than that of Ni^2+^ oxidation probably due to the hole accumulation.

The superior performance of the Fe@Ni–MOF/Fe_2_O_3_:Ti can be further certified by open‐circuit potential. As shown in Figure S19 (Supporting Information), the Fe@Ni–MOF/Fe_2_O_3_:Ti photoanode demonstrats the lowest open‐circuit photovoltage among the measured samples. On the basis of the relation (11) of open‐circuit photovoltage (*V*_ph_), the onset potential (*E*_onset‐light_), and the kinetic overpotential (*ξ*), the Fe@Ni–MOF/Fe_2_O_3_:Ti processes the lowest kinetic overpotential

(11) $$\begin{array}{c} V_{\mathrm{ph}}=E_{\mathrm{redox}}+\xi -E_{\mathrm{onset}-\mathrm{light}} \end{array}$$

From the above discussion, it is confirmed that the Fe@Ni–MOF cocatalyst can open a new pathway for efficiently promoting hole transfer and significantly reducing the interfacial charge recombination, resulting in the excellent PEC OER of the Fe_2_O_3_:Ti photoanode.

## 3 Conclusion

In summary, the Fe@Ni–MOF shell was formed on the surface of the Fe_2_O_3_:Ti nanorods via a facile one‐step solvothermal process. As a proof‐of‐concept demonstration, a promoting effect toward PEC water oxidation is achieved by the Fe incorporation into the Ni–MOF. Optimal PEC water oxidation performance can be realized by tuning the molar ratio of Fe:Ni. Specifically speaking, the Fe@Ni–MOF(Fe:Ni = 1:10)/Fe_2_O_3_:Ti photoelectrode demonstrated a dramatic enhancement in photocurrent density (2.3 ± 0.06 mA cm^−2^ at 1.23 V vs RHE) and a quite low onset potential (780 mV vs RHE). Additionally, we investigated the unique difference in the PEC water performance of Ni–MOF and Fe@Ni–MOF modified Fe_2_O_3_:Ti photoanodes. For the Ni–MOF/Fe_2_O_3_:Ti photoanode, sluggish charge transfer rate (*K*_ct_) results in smaller charge transfer efficiency, which is unfavorable for PEC OER. Instead, incorporating Fe into Ni–MOF inhibits surface charge recombination (*K*_rec_), which improves the charge transfer efficiency. The difference in PEC performance mainly derives from the bimetal interaction. From the XPS results, the interaction of both Ni and Fe may be able to optimize the d‐orbital filling and charge transfer ability, which can improve the catalytic activity. Additionally, we also reveal that the introduced Fe can donate electrons to Ni in the MOF structure due to a high electronegativity of Ni under the oxidized bias > ∼1.4 V. The work has illuminated the importance of charge transfer efficiency on the final PEC performance and contributes to understand the mutual effect between a semiconductor and MOF electrocatalyst. Future research will focus on the interface of semiconductor‐catalyst junctions and provide more guidelines for designing effective complex photoanodes.

## Conflict of Interest

The authors declare no conflict of interest.

## Supporting information

Supporting Information
