# Supplementary material for: Modulating Charge Transfer Efficiency of Hematite Photoanode with Hybrid Dual‐Metal–Organic Frameworks for Boosting Photoelectrochemical Water Oxidation
Source: Adv Sci (Weinh). 2020 Oct 25;7(23):2002563. doi: 10.1002/advs.202002563 (PMC7709986; doi:10.1002/advs.202002563)
Supplement: Supplementary file 1 — Supporting Information [file ADVS-7-2002563-s001.pdf]

WILEY-VCH

© 2020 Wiley-VCH GmbH

ADVANCED SCIENCE
Open Access

Supporting Information

for *Adv. Sci.*, DOI: 10.1002/advs.202002563

Modulating Charge Transfer Efficiency of Hematite Photoanode with Hybrid Dual-Metal-Organic Frameworks for Boosting Photoelectrochemical Water Oxidation

Keke Wang, Yang Liu, Kenta Kawashima, Xuetao Yang, Xiang Yin, Faqi Zhan, Min Liu, Xiaoqing Qiu, Wenzhang Li, C. Buddie Mullins* and Jie Li**

Supporting Information

Modulating Charge Transfer Efficiency of Hematite Photoanode with Hybrid Dual-Metal-Organic Frameworks for Boosting Photoelectrochemical Water Oxidation

Keke Wang, Yang Liu, Kenta Kawashima, Xuetao Yang, Xiang Yin, Faqi Zhan, Min Liu, Xiaoqing Qiu, Wenzhang Li, C. Buddie Mullins* and Jie Li**

Dr. K. Wang, Dr.Y. Liu, Dr. X. Yang, Dr. X. Yin, Dr. F. Zhan, Prof. X. Qiu, Prof. W. Li, Prof. J. Li
School of Chemistry and Chemical Engineering, Central South University, Changsha 410083 China.
E-mail: liwenzhang@csu.edu.cn (W. Li); lijieliu@csu.edu.cn (J. Li)

Prof. M. Liu
Institute of Super-microstructure and Ultrafast Process in Advanced Materials, School of Physics and Electronics, Central South University, Changsha 410083,China

Mr. K. Kawashima, Prof. C. B. Mullins
McKetta Department of Chemical Engineering and Department of Chemistry, University of Texas at Austin, Austin, TX, 78712-0231, United States
E-mail: mullins@che.utexas.edu (C. B. Mullins)

Prof. W. Li
Hunan Provincial Key Laboratory of Efficient and Clean Utilization of Manganese Resources, Central South University, Changsha 410083,China

Experimental Details

Preparation of the TiO_2 underlayers. Fluorine-doped tin oxide (FTO) coated glass slides (40 × 30 × 1.7 mm) were ultrasonically washed with acetone, isopropanol alcohol, ethanol, and deionized water bath for 30 min in turn. TiO_2 compact layer was coated onto the cleaned FTO glasses by a spin-coating method (3000 rpm for 30 s), using $TiCl_4$ solution as precursor. In the end, the as-prepared samples were heated at 500 °C for 30 min.

Preparation of Ti-doped Fe_2O_3 nanorod arrays. The preparation of Ti-doped Fe_2O_3 nanorod array on TiO_2-coated FTO substrates following a similar hydrothermal procedure as reported previously[1]. Typically, 0.15 M $FeCl_3{\cdot}6H_2O$ and 1 M $NaNO_3$ were dissolved in deionized water, and then hydrochloric acid was added into the solution under magnetic stirring to adjust the pH value to be around 1.25. The as-prepared FTO substrate was tilt placed in an autoclave with the conducting side facing down and the above solution was

transferred to the autoclave. The hydrothermal reaction was held at 120 °C for 4 h in an air oven. Then the film was washed several times with deionized water and dried. Finally, the samples were annealed in a tube furnace at 550 °C for 2 h and further at 700 °C for 10 min in air to produce the Ti-doped Fe_2O_3 nanorod arrays (referred to Fe_2O_3:Ti).

Preparation of Ni-MOF/Fe_2O_3:Ti, Fe-MOF/Fe_2O_3:Ti, and Fe@Ni-MOF/Fe_2O_3:Ti composites. A Ni-MOF/Fe_2O_3:Ti sample was prepared via a facile solvothermal method. Briefly, 0.172 g of $Ni(NO_3)_2{\cdot}6H_2O$, 0.082 g of 1,3,5-trimesic acid (BTC), and 0.022g of 2-methyl-imidazole were added into 30 mL of N, N-dimethylformamide (DMF) solution under magnetic stirring. Afterwards, the mixture was transferred into the autoclave with the as-synthesized Fe_2O_3:Ti films inside. Subsequently, the hydrothermal reaction was proceeded at 170 °C for 48 h in an air oven. Finally, the composite photoelectrode material (denoted as Ni-MOF/Fe_2O_3:Ti) was washed with DMF and ethanol several times, and then dried in vacuum at 80 °C for 12 h in vacuum.

Fe-MOF/Fe_2O_3:Ti and Fe@Ni-MOF/Fe_2O_3:Ti photoanode were fabricated with the similar method of the Ni-MOF/Fe_2O_3:Ti except adding $Fe(NO_3)_3{\cdot}9H_2O$ and the different amount of $Fe(NO_3)_3{\cdot}9H_2O$. According to the atomic ratio of Fe/Ni, the corresponding samples were labeled as Fe@Ni-MOF(1:1)/Fe_2O_3:Ti, Fe@Ni-MOF(1:5)/Fe_2O_3:Ti, Fe@Ni-MOF(1:10)/Fe_2O_3:Ti, and Fe@Ni-MOF(1:15)/Fe_2O_3:Ti, respectively.

Physical characterization. The crystalline structures of all films were identified by X-ray diffraction (XRD, D/Max2250, Rigaku) using Cu Kα radiation (λ =0.15406 nm). The recorded diffraction peak positions were calibrated by using a SnO_2 (110) diffraction peak at 26.578°. The morphologies of the as-prepared composites were observed by a scanning electron microscope (SEM, Nova NanoSEM 230, FEI) and high-resolution transmission electron microscope (HRTEM, Tecnai G2 F20, FEI). The energy dispersive X-ray spectroscopy (EDX) was used for elemental analyses with the accelerating voltage of 20 kV.

The surface composition and elemental valence state were examined by using an X-ray photoelectron spectrometer (XPS, K-Alpha 1063, Thermo Scientific). Incidentally, this system utilized 50.00 eV pass energy. The band energies were calibrated with respect to the residual C 1s peaks. The UV-vis absorption spectra were recorded through a diffuse reflectance-ultraviolet (DR-UV) spectrophotometer (UV-2450, Shimadzu). The resultant composite structures were analyzed by a Fourier Transform Infrared (FT-IR) spectrometer (Nicolet 6700, Thermo Scientific).

Photoelectrochemical measurements. All the photoelectrochemical (PEC) tests were carried out at room temperature by using an electrochemical workstation with a three-electrode configuration under simulated AM 1.5G illumination (100 Mw cm^{-2}). In this setup, the as-prepared photoanode, a platinum plate, a Hg/HgO electrode, and 1.0 M KOH (pH = 13.4) were used as the working electrode, counter electrode, reference electrode, and electrolyte, respectively. All the as-prepared photoanodes were illuminated from the back side. The photocurrent curve was obtained at a scanning rate of 20 mV/s. Transient photocurrent (TP) spectroscopy was conducted with the same PEC setup, in which the data acquisition sets as 0.1 s. The electrochemical impedance spectroscopy (EIS) was carried out in the potential range of 0.5-1.3 V *vs*. the reversible hydrogen electrode (RHE) with an AC frequency ranging from 10 kHz to 100 mHz and analyzed by Z-View program (Scribner Associates, Inc.). The Mott−Schottky measurements were conducted with an AC frequency of 1 kHz the illumination condition. The incident photon-to-current conversion efficiency (IPCE) tests were performed with a xenon lamp (150 W, Oriel) equipped with a monochromator at a bias of 1.23 V (*vs*. RHE). Intensity modulated photocurrent spectrum (IMPS) was performed at the varying potentials from 0.5 to 1.3 V (*vs*. RHE) by a Zahner CIMPS system with the frequency ranging from 1 kHz to 0.1 Hz, in which a white light emitting diode lamp was served as light source. The evolved H_2 and O_2 gases were collected and characterized by a gas chromatograph (GC-5890N, Agilent) equipped with a thermal conductivity detector (TCD).

To eliminate the ambient air existing in the reactor, N_2 gas was aerated into the reactor for 30 min before the tests. All the recorded potentials were converted to the potential of RHE using the Nernst equation: $E_{RHE} = E_{Hg/HgO} + E^{\theta}_{Hg/HgO} + 0.059 \times pH$.

The direct current (dc) cyclic voltammograms and Fourier transformed (FT) alternating current (ac) voltammograms measurements were carried out in a CHI760e electrochemical workstation. Then, the ac voltammetric experiments were measured with amplitude of 0.05 V, which provides an adequate level of nonlinearity to obtain higher order harmonics to be detected (fitted by systematic software CHI760e), and in the meantime, does not induce very significant ohmic losses and broadening. The test frequency was 9 Hz, which provides sufficient level of kinetic sensitivity[2].

Statistical Analysis

1. The diameter distribution statics of different photoanodes (Figure S2) was calculated by Image J software. Then these datas were imported into the the Origin software. Finally, the plots and corresponding mean and standard deviation were obtained. The calculation particles sizes for different photoanodes were 54.2 ± 11.3, 67.2 ± 14.8, 72.7 ± 18.1 and 64.6 ± 10.6 nm, respectively.

2. The high-resolution XPS spectra were fitted by the XPSPEAK41 software.

3. With regard to the calculation of photocurrent density (*J*) at 1.23 V vs. RHE (Figure S11), mean and standard deviation of photocurrent density for Fe_2O_3:Ti and Fe@Ni-MOF/Fe_2O_3:Ti photoanodes were calculated. *J* (Fe_2O_3:Ti) = 0.76 ± 0.02 mA cm^{-2}. *J* (Fe@Ni-MOF/Fe_2O_3:Ti) = 2.3 ± 0.06 mA cm^{-2}.

4. The applied bias photon-to-current efficiency (ABPE) in Figure 3c can be obtained by the following formula (1). Finally, the ABPE was plotted by using the Origin software.

$$\mathrm{ABPE} = \mathrm{J} \times \frac{1.23 - E_{RHE}}{P} \times 100\% \tag{1}$$

where J represents the photocurrent density ($mA \cdot cm^{-2}$) at the applied bias V *vs*. RHE, E_{RHE} is the applied bias at the reversible hydrogen electrode (RHE) condition, P is the incident light intensity ($mW \cdot cm^{-2}$).

5. The Bluter plots (Figure 3b) were obtained by taking the square of photocurrent density (J^2) as Y-axis and Potential (V vs. RHE) as X-axis. Then, linear fitting was adopted in an Origin software.

6. Calculation of the incident-photon-to-current conversion efficiency (IPCE) in Figure 3d according to the equation (2). Finally, the ABPE was plotted by using the Origin software.

$$\mathrm{IPCE}(\%) = (1240 \times \mathrm{J})/(\lambda \times P) \quad (2)$$

Where J is the photocurrent density ($mA \cdot cm^{-2}$) at the applied bias V *vs*. RHE, λ is the wavelength of incident light (nm), P is the incident light intensity ($mW \cdot cm^{-2}$).

7. The charge separation efficiency (η_{inj}) and charge injection efficiency (η_{sep}) in Figures 3e and 3f were calculated by the following equation (3) and (4).

$$\eta_{inj} = J_{\mathrm{H_2O}}/J_{\mathrm{Na_2so_3}} \quad (3)$$

$$\eta_{\mathrm{sep}} = J_{\mathrm{Na_2SO_3}}/\mathrm{J_{abs}} \quad (4)$$

where $J_{\mathrm{H_2O}}$ represents the photocurrent density without adding Na_2SO_3 hole scavenger, $J_{Na_2so_3}$ is the photocurrent density with adding Na_2SO_3 hole scavenger, J_{abs} is determined to be 12.3 $mA \cdot cm^{-2}$ by integrating the UV-vis absorption spectra of photoanodes films with respect to the AM 1.5 G solar light spectrum.

8. The PEIS Nyquist plots in Figure 4 were fitted by the Equivalent circuit (Figure S14) in the ZView software.

9. For the charge transfer (K_{ct}) and charge recombination (K_{rec}) in Figure 5, they can be obtained by the equations (1)-(4) in the manuscript. Then, the corresponding plots were plotted using the Origin software. The charge transfer efficiency (φ_{ct}) in Figure 6 can be calculated by the equations (5) and (6) in the manuscript.

10. To calculated the elecrochemically active surface areas (ECSAs) of samples, a linear trend was obtained by plotting the difference between the anodic and cathodic current densities against the scan rate at 0.7 V *vs.* RHE by using the Origin software. The R^2 (statistics) of the fitting line for the Fe_2O_3:Ti, Ni-MOF/Fe_2O_3:Ti, Fe@Ni-MOF/Fe_2O_3:Ti and Fe-MOF/Fe_2O_3:Ti are 0.9995, 0.9990, 0.9985, and 0.9999, respectively. The corresponding C_{dl} values were calculated to be 0.05 ± 0.0005, 0.11± 0.0018, 0.13±0.0025 and 0.08 ± 0.0004 mF cm^{-2} for the Fe_2O_3:Ti, Fe-MOF/Fe_2O_3:Ti, Ni-MOF/Fe_2O_3:Ti, and Fe@Ni-MOF(Fe:Ni = 1:10)/Fe_2O_3:Ti. The calculated ECSAs of the Fe_2O_3:Ti, Ni-MOF/Fe_2O_3:Ti, Fe@Ni-MOF/Fe_2O_3:Ti and Fe-MOF/Fe_2O_3:Ti samples are 1.08 ± 0.012, 2.78± 0.045, 3.25± 0.063, and 1.99± 0.010 cm^2, respectively according to the equations (5) and (6).

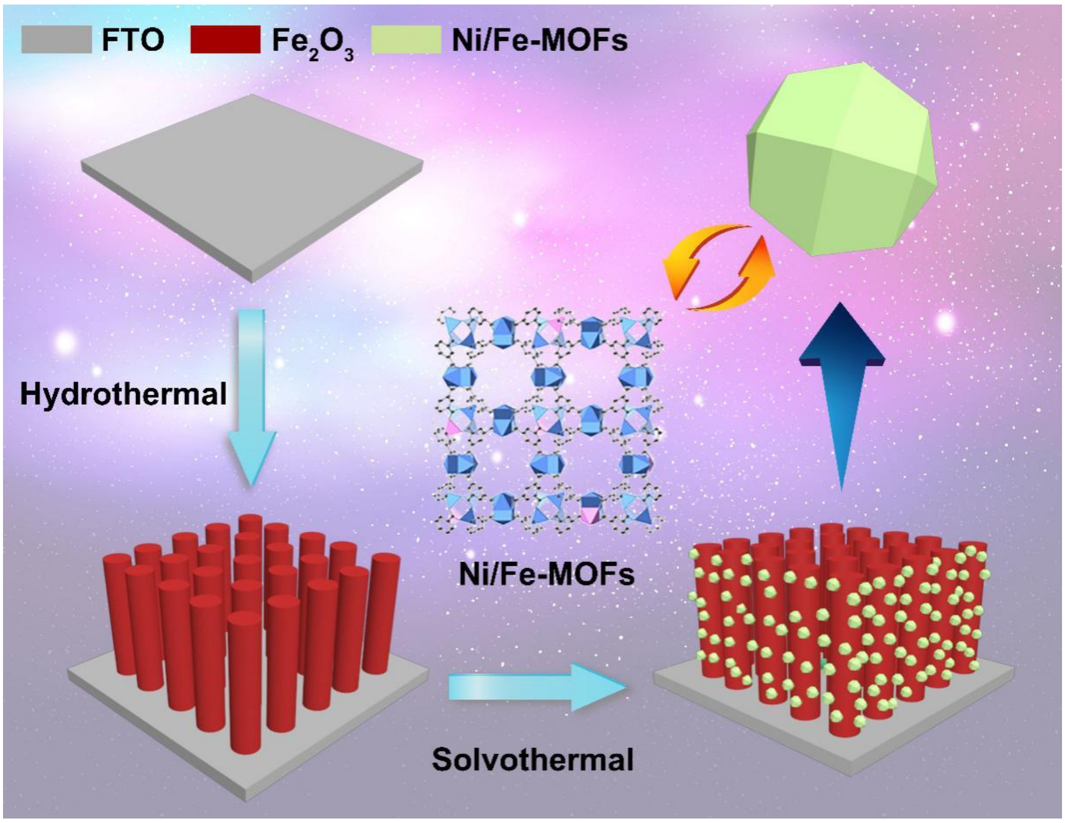

Figure S1. Schematic diagram of Fe@Ni-MOF/Fe_2O_3:Ti photoanode fabrication for PEC water oxidation.

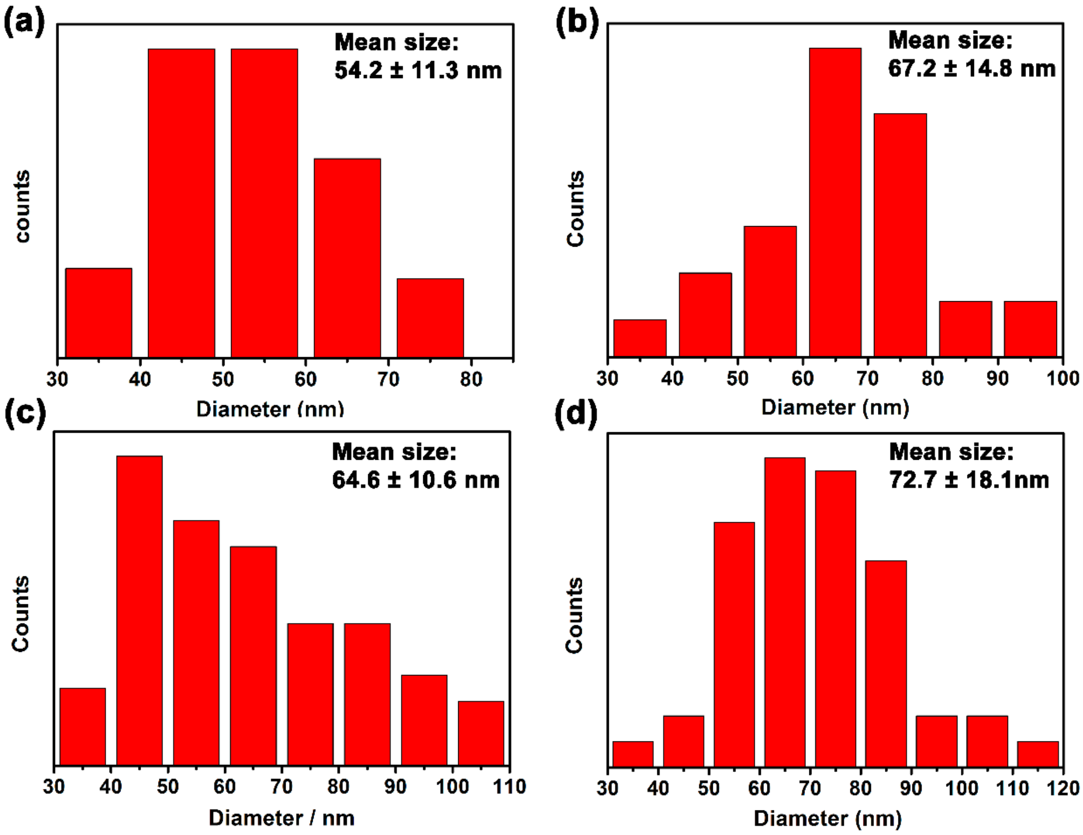

Figure S2. Diameter distribution statics of different photoanodes: a) Fe_2O_3:Ti, b) Ni-MOF/Fe_2O_3:Ti, c) Fe@Ni-MOF (Fe:Ni= 1:10)/Fe_2O_3:Ti, and d) Fe-MOF/Fe_2O_3:Ti.

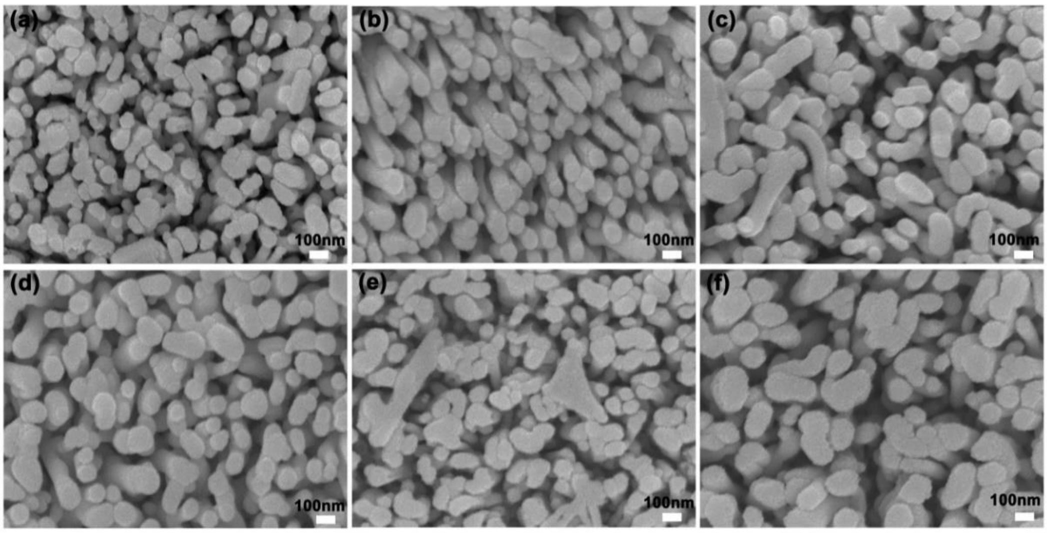

Figure S3. Top-view SEM images of a) Ni-MOF/Fe_2O_3:Ti, b) Fe@Ni-MOF (Fe:Ni = 1:1)/Fe_2O_3:Ti, c) Fe@Ni-MOF (1:5)/Fe_2O_3:Ti, d) Fe@Ni-MOF(1:10)/Fe_2O_3:Ti, e) Fe@Ni-MOF(1:15)/Fe_2O_3:Ti, and f) Fe-MOF/Fe_2O_3:Ti photoanodes.

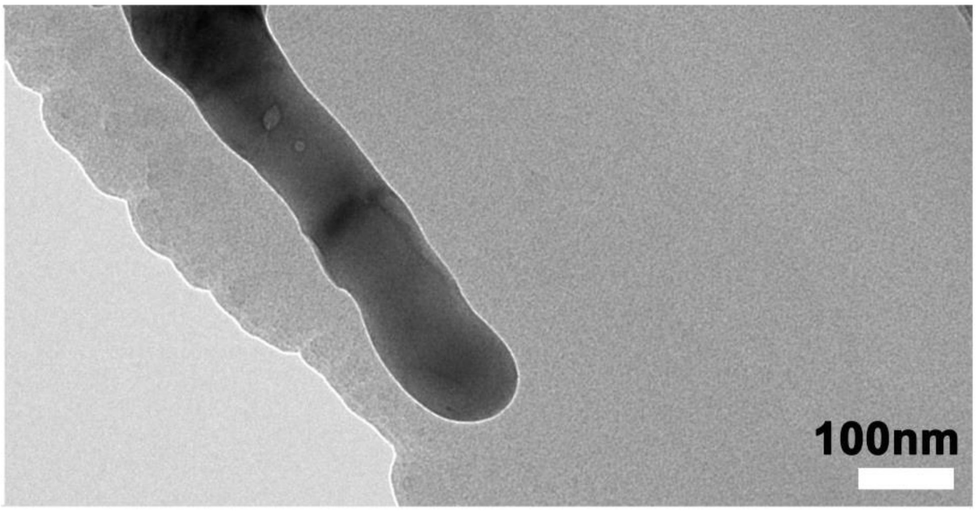

Figure S4. TEM image of Fe_2O_3:Ti nanorods.

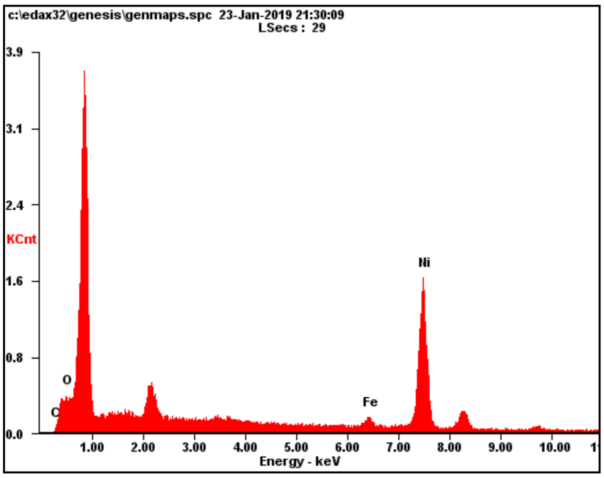

Element	*Wt%*	*At%*
**C K**	03.14	11.44
**O K**	08.22	22.45
**Fe K**	02.99	02.34
**Ni K**	85.65	63.77
**Matrix**	Correction	ZAF

Figure S5. Energy-dispersive X-ray spectroscopy (EDX) spectrum and elemental analysis for Fe@Ni-MOF (Fe:Ni =1:10)/Fe_2O_3:Ti photoanode.

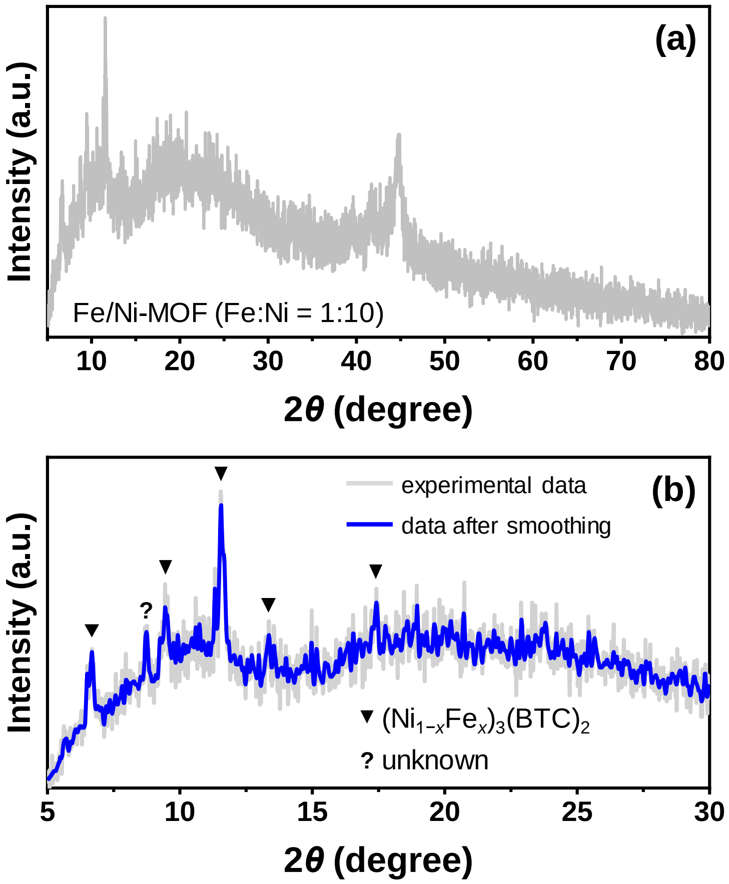

Figure S6. XRD patterns of Fe@Ni-MOF powder sample (Fe:Ni = 1:10) over a) wide and b) narrow 2-theta ranges: 5-80° and 5-30°, respectively.

In the XRD patterns of the MOF-loaded Fe2O3:Ti samples (see Figure 2a), a few unknown diffraction peaks with weak peak intensities were also confirmed. Specifically, the diffraction peaks at around 22° are not existed in the reference[3]. However, a similar peak was also found in other metal-BTC-based MOFs, such as Ni-MOF[4] and La-BTC[5]. Thus, there is a possibility that the as-observed unknown diffraction peaks also correspond to Ni-, Fe-, and Fe@Ni-BTC MOF.

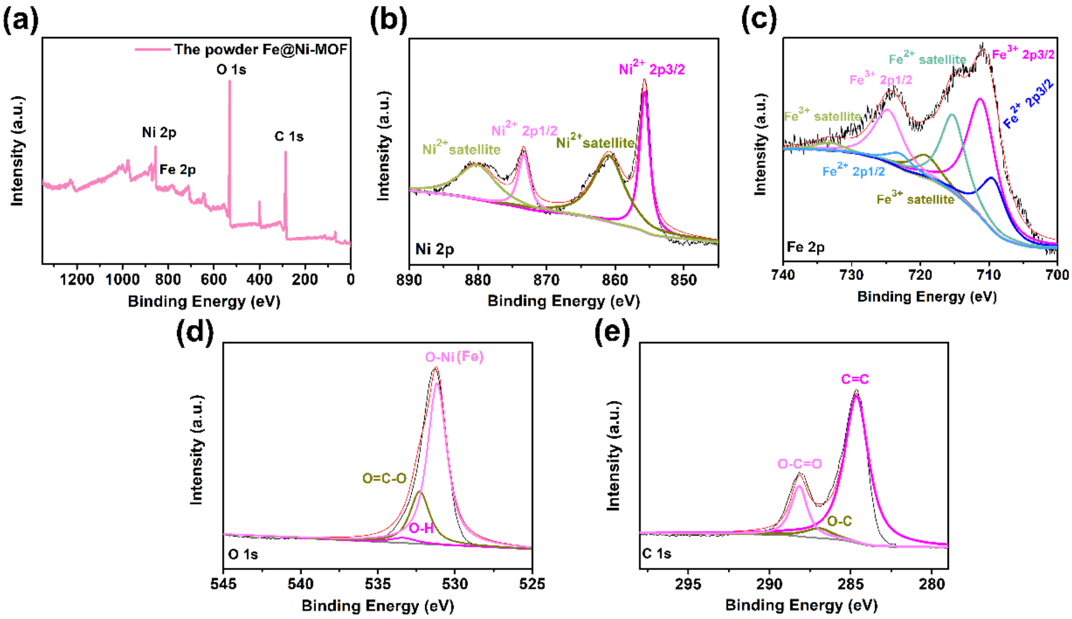

Figure S7. Powder XPS spectra of Fe@Ni-MOF (Fe:Ni =1:10). a) Survey scan, b) Ni 2p, c) Fe 2p, d) O 1s, and e) C 1s spectra.

The XPS survey spectrum of the Fe@Ni-MOF (Fe:Ni = 1:10) indicates the coexistence of Fe, Ni, C, and O elements (Figure S7a). The high-resolution Ni 2p spectrum demonstrates the presence of Ni^{2+} (Figure S7b), where the peak can be deconvoluted into Ni $2p_{3/2}$ and Ni $2p_{1/2}$ at the binding energy of 855.7 and 873.3 eV, respectively. Furthermore, Fe 2p spectrum exits two main peaks at around 711.7 (Fe $2p_{3/2}$) and 724.0 eV (Fe $2p_{1/2}$), which are the characteristic peaks of Fe^{3+}. Additionally, the small Fe^{2+} peaks can also be found in Fe@Ni-MOF, which matches a previous report[6] (Figure S7c). For the O 1s spectrum (Figure S7d), the peak at around 531.3 eV is attributed to the Ni(Fe)-O bond, while another two peaks located in the higher banding energy can ascribed to the O=C-O and O-H on the trimesic linkers and possibly absorbed water molecules. Figure S7e shows the spectrum of C 1s, which can be fitted by three peaks at around 284.7, 286.9, and 288.2 eV related to the benzoic rings (C=C), the carboxylate (O-C=O) group, and the C-O bond, respectively.

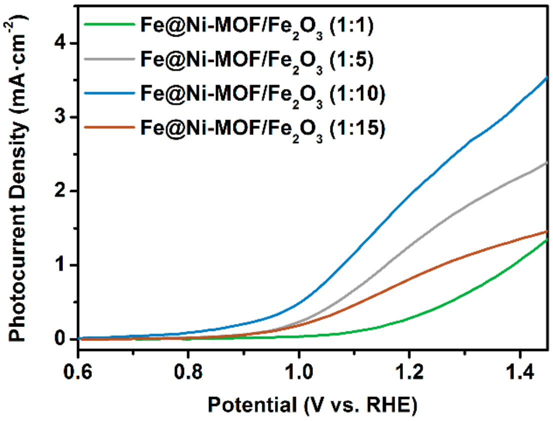

Figure S8. *J-V* curves for Fe@Ni-MOF/Fe_2O_3:Ti composites with different ratios of Fe/Ni.

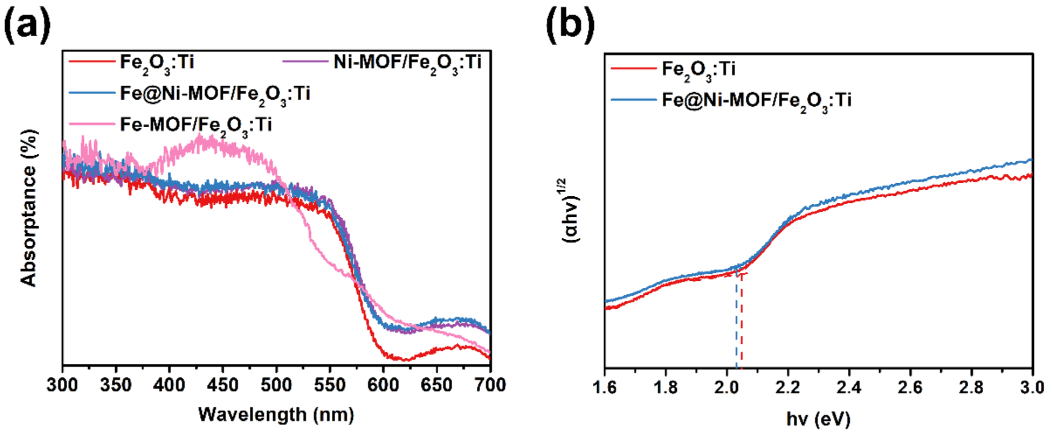

Figure S9. a) UV-vis absorption spectra of Fe_2O_3:Ti, Ni-MOF/Fe_2O_3, Fe-MOF/Fe_2O_3:Ti, and Fe@Ni-MOF(Fe:Ni = 1:10)/Fe_2O_3:Ti photoanodes, b) Tauc plots of Fe_2O_3:Ti and Fe@Ni-MOF(Fe:Ni = 1:10)/Fe_2O_3:Ti photoanodes.

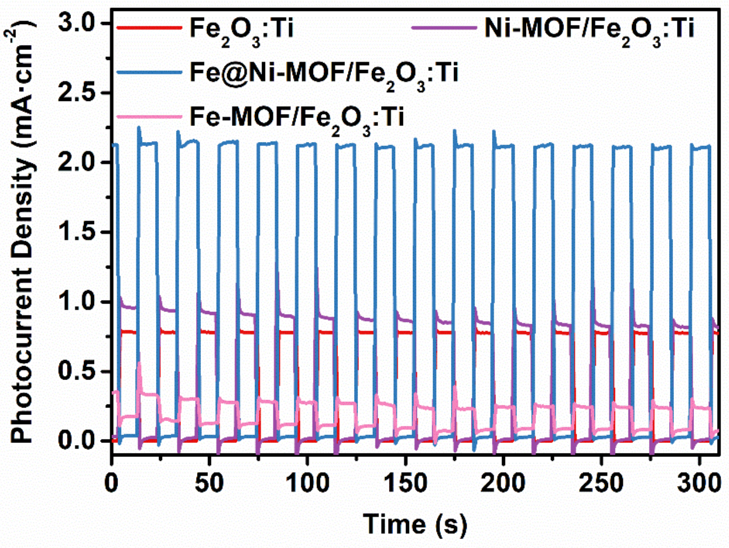

Figure S10. *I-t* curves of Fe_2O_3:Ti, Ni-MOF/Fe_2O_3 Fe_2O_3:Ti, Fe@Ni-MOF (Fe:Ni = 1:10)/Fe_2O_3:Ti, and Fe-MOF/Fe_2O_3:Ti photoanodes at 1.23 V *vs*. RHE under chopped simulated sunlight.

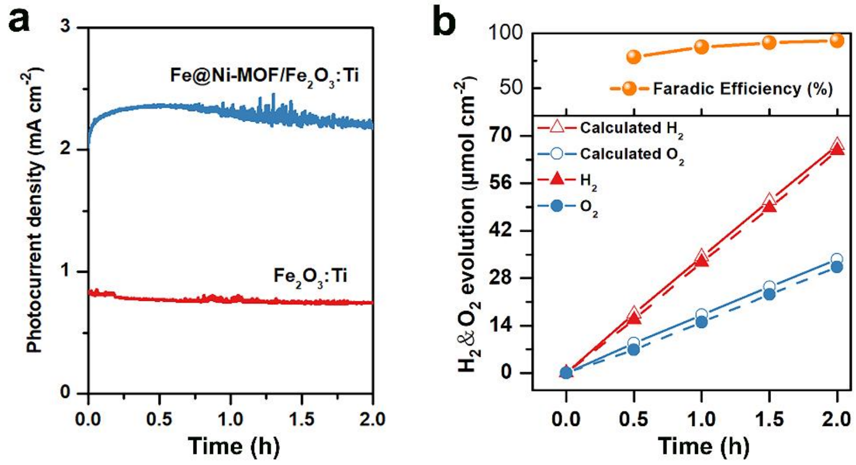

Figure S11. a) Photostability test results of Fe_2O_3:Ti and Fe@Ni-MOF(Fe:Ni = 1:10)/Fe_2O_3:Ti photoanodes at 1.23 V *vs*. RHE. b) Reaction time courses for PEC H_2 and O_2 evolution over Fe@Ni-MOF/Fe_2O_3:Ti photoanode at 1.23 V *vs*. RHE and the corresponding faradic efficiency for PEC O_2 evolution.

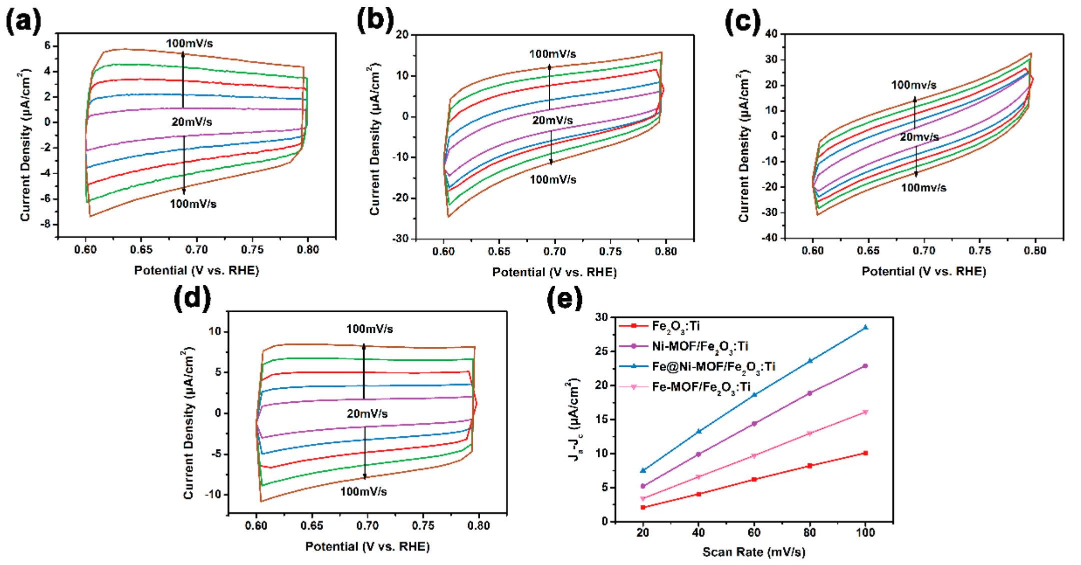

Figure S12. Cyclic voltammetry (CV) curves of a) bare Fe_2O_3:Ti, b) Ni-MOF/Fe_2O_3:Ti, c) Fe@Ni-MOF(Fe:Ni =1:10)/Fe_2O_3:Ti, and d) Fe-MOF/Fe_2O_3:Ti. e) Current density difference at 0.7 V *vs*. RHE plotted against scan rates in a non-Faradaic region. (The R^2 of the fitting line for the Fe_2O_3:Ti, Ni-MOF/Fe_2O_3:Ti, Fe@Ni-MOF/Fe_2O_3:Ti and Fe-MOF/Fe_2O_3:Ti are 0.9995, 0.9990, 0.9985, and 0.9999, respectively.)

The elecrochemically active surface areas (ECSAs) of the as-prepared photoanodes were estimated according to the double-layer capacitance (C_{dl}), which is based on cyclic voltammetry (CV) measurements at a non-Faradaic potential region. A series of CV tests were carried out at different scan rates (20, 40, 60, 80, and 100 mV/s) within 0.6-0.8 V *vs*. RHE (Figures. S12a-d). A linear trend was obtained by plotting the difference between the anodic and cathodic current densities against the scan rate at 0.7 V *vs*. RHE (Figure S12e). Thereinto, the double-layer capacitance (C_{dl}) values can be obtained according to the following formula (1):

$$I_c = C_{dl}\frac{dV}{dt} \tag{5}$$

The corresponding C_{dl} values were calculated to be 0.05 ± 0.0005, 0.11± 0.0018, 0.13±0.0025 and 0.08 ± 0.0004 mF cm^{-2} for the Fe_2O_3:Ti, Fe-MOF/Fe_2O_3:Ti, Ni-MOF/Fe_2O_3:Ti, and Fe@Ni-MOF(Fe:Ni = 1:10)/Fe_2O_3:Ti. As expected, the Fe@Ni-MOF/Fe_2O_3:Ti photoanode exhibited the largest C_{dl} value among all the measured

photoanodes, indicating that the presence of the Fe@Ni-MOF can provide a large active area and expose more active sites at the interface of photoanode/electrolyte. Additionally, the renormalized photocurrent density-potential (J-V) curves based on ECSA were plotted to evaluate the intrinsic PEC water oxidation performance. The ECSAs of the different photoanodes were obtained by the equation (2):

$$\text{ECSA} = \frac{C_{dl}}{C_s} \tag{6}$$

where C_s refers to the specific capacitance of the sample[7].

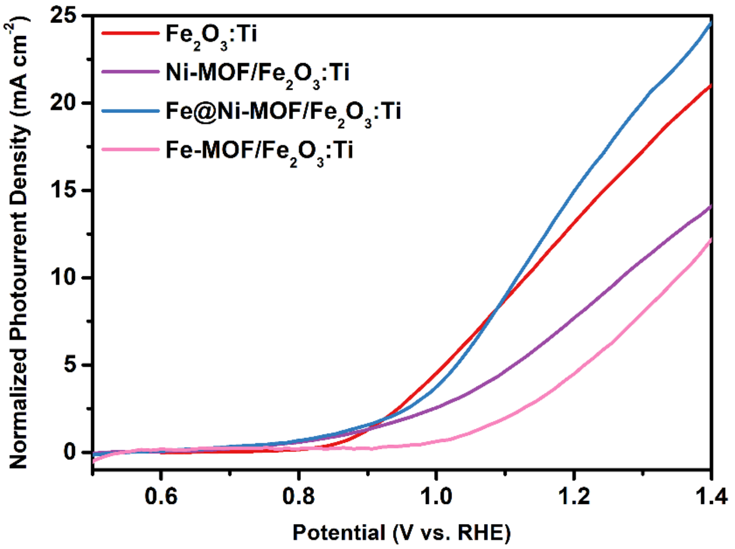

Figure S13. Photocurrent density versus applied potential curves (based on ECSA) of Fe_2O_3:Ti, Ni-MOF/Fe_2O_3:Ti, Fe@Ni-MOF(Fe:Ni = 1:10)/Fe_2O_3:Ti, and Fe-MOF/Fe_2O_3:Ti photoanodes.

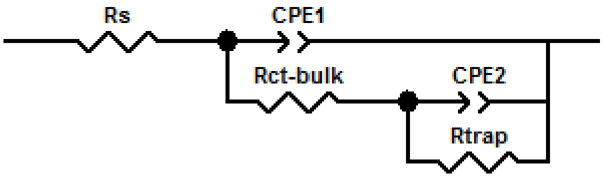

Figure S14. Equivalent circuit for explanation of Fe_2O_3:Ti and MOFs modified Fe_2O_3:Ti photoanodes.

R_s represents a resistance ascribable to electrolyte. CPE1 is a bulk capacitor relevant to the space charge region. $R_{ct,bulk}$ is considered as a charge transfer resistance in the bulk hematite photoanode. $R_{ct,trap}$ defines as a charge transfer resistance across the electrolyte/hematite interface. CPE2 is according to the surface states capacitor. To calculate the capacitance, the CPE component was fit with CPE-P and CPT-T components in the following equation:

$$C_{\mathrm{eff}} = \left[Q\left(\frac{1}{R_{\mathrm{s}}} + \frac{1}{R_{trap}}\right)^{(\alpha-1)}\right]^{1/\alpha} \quad (7)$$

where CPE-P represents α, CPE-T is Q, R_s is solution resistance, and R_t is charge transfer resistance[8].

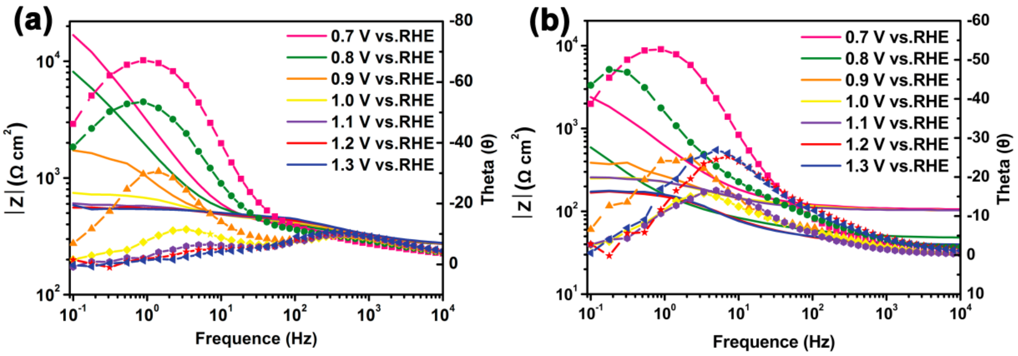

Figure S15. Derived Bode plots of a) Fe_2O_3:Ti and b) Fe@Ni-MOF(Fe:Ni = 1:10)/Fe_2O_3:Ti photoanodes at different potentials.

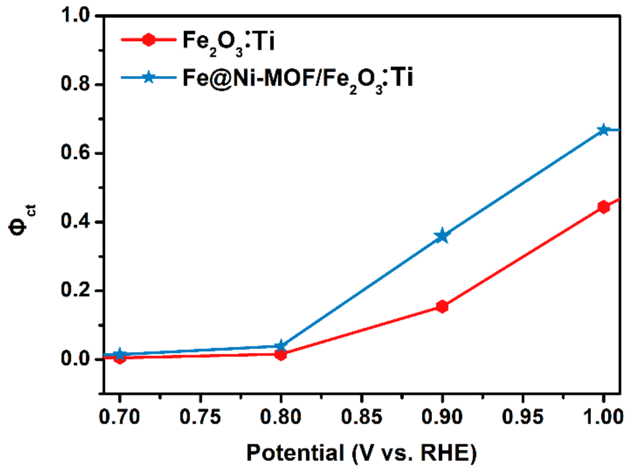

Figure S16. Charge transfer efficiency of Fe_2O_3:Ti and Fe@Ni-MOF (Fe:Ni = 1:10)/Fe_2O_3:Ti photoanodes.

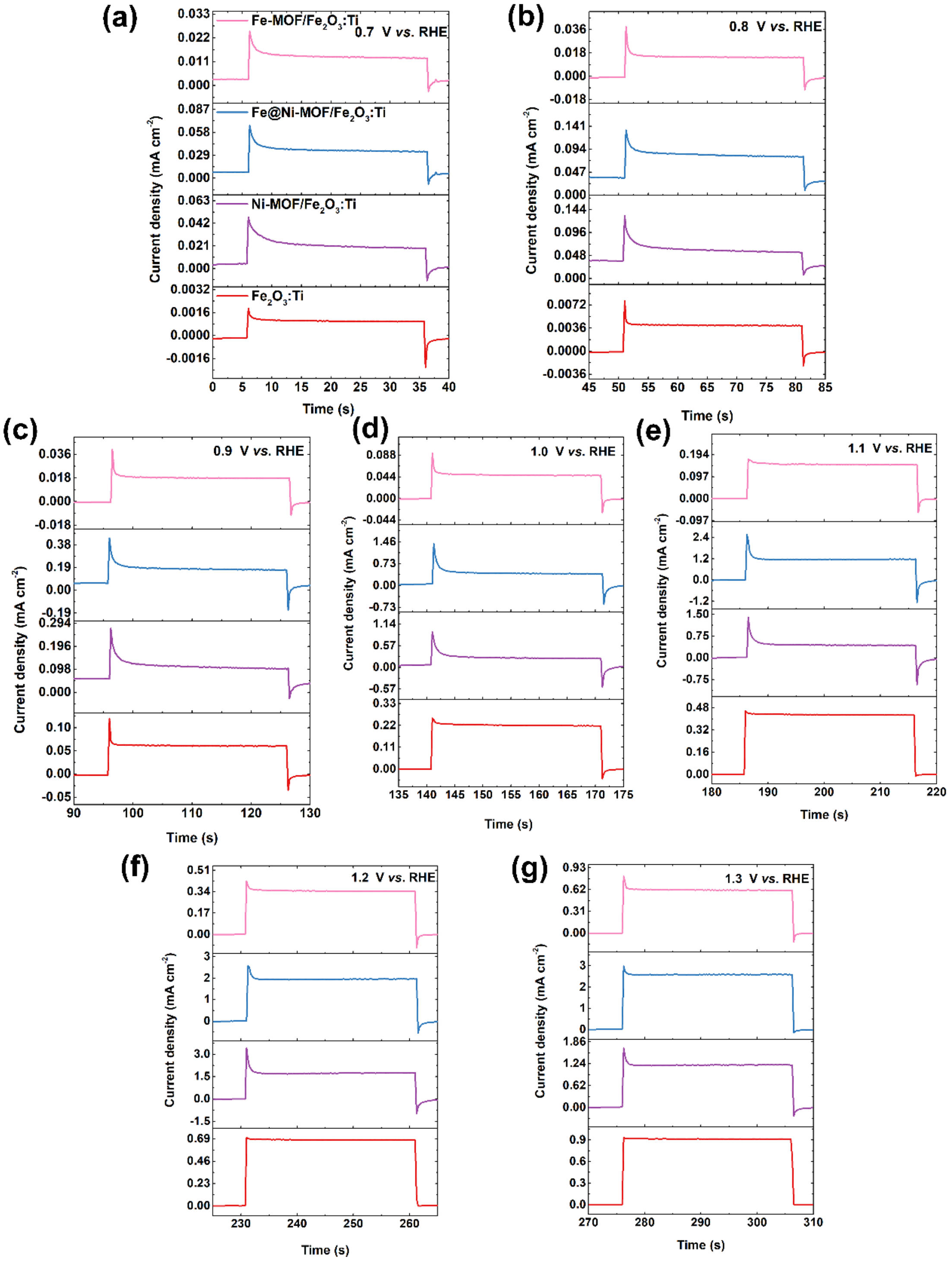

Figure S17. Transient photocurrent of pristine Fe_2O_3:Ti and MOFs-modified Fe_2O_3:Ti photoanodes at different potentials. Here, the molar ratio of Fe:Ni in Fe@Ni-MOF is 1:10.

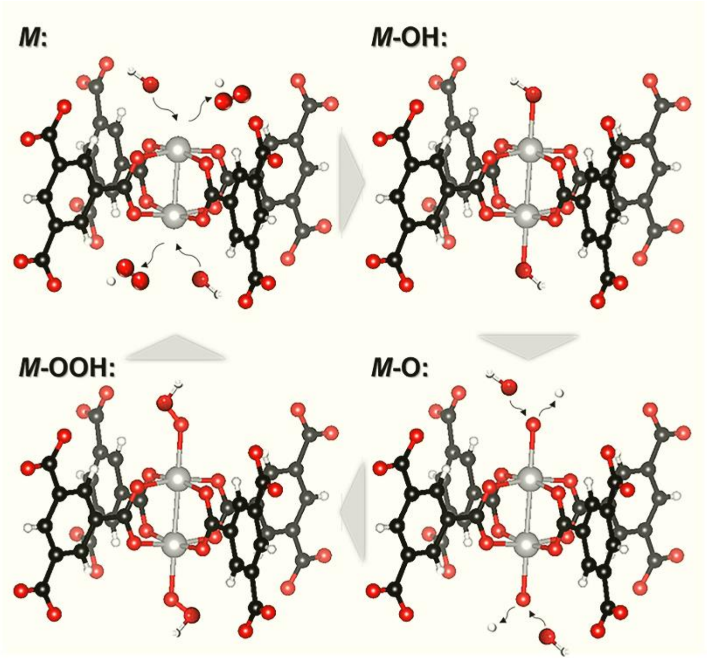

Figure S18. Schematic representation of OER mechanism of *M*-MOF (*silver*: transition metal; *red*: oxygen; *black*: carbon; *white*: hydrogen).

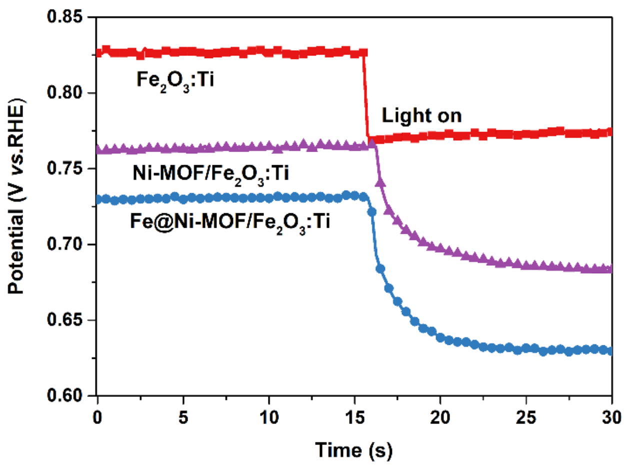

Figure S19. Open circuit potential shifts of different photoanodes: Fe_2O_3:Ti, Fe@Ni-MOF (Fe:Ni = 1:10)/Fe_2O_3:Ti, and Ni-MOF/Fe_2O_3:Ti.

Table S1. A comparision of the Fe@Ni-MOF(Fe:Ni = 1:10)/Fe_2O_3:Ti photoanode in this work with previously reported Fe_2O_3-based photoanodes for PEC water oxidation in alkaline electrolyte.

Co-catalyst	Preparation method	Testing conditions	Photocurrent density at 1.23 V_{RHE} (mA cm^{-2})	Reference
Co_3O_4	Hydrothermal	1 M NaOH	1.20	[9]
FeCoW	Spin-coating	1 M NaOH	1.18	[10]
FeOOH	Solution-based precipitation	1 M NaOH	1.21	[11]
NiFe phosphate	Dip-coating	0.1 M KOH	1.2	[12]
MnO_2	Dip-coating	1 M KOH	0.68	[13]
NiOOH	Photoelectrodeposited	1 M NaOH	0.625	[14]
C nanodots and Co_3O_4 nanoclusters	Electrochemical and hydrothermal	1 M NaOH	1.48	[15]
MIL-101(Fe)<br>NH2-MIL-101	Solverthermal	1 M NaOH	1.26<br>2.27	[16]
Fe@Ni-MOF	Solverthermal	1 M KOH	2.1	This work

References

[1] A. Annamalai, P. S. Shinde, A. Subramanian, J. Y. Kim, J. H. Kim, S. H. Choi, J. S. Lee, and J. S. Jang, *J. Mater. Chem. A* 2015, 3, 5007.
[2] A. M. Bond, D. Elton, S.-X. Guo, G. F. Kennedy, E. Mashkina, A. N. Simonov, and J. Zhang, *J. Electrochem. Commun.* 2015, 57, 78.
[3] C. R. Wade, and M. Dincă, *Dalton Trans.* 2012, 41, 7931.
[4] V. Maruthapandian, S. Kumaraguru, S. Mohan, V. Saraswathy, and S. Muralidharan, *ChemElectroChem* 2018, 5, 2795.
[5] S. R. Vaddipalli, S. R. Sanivarapu, S. Vengatesan, J. B. Lawrence, M. Eashwar, and G. Sreedhar, *ACS Appl. Mater. Interfaces* 2016, 8, 23049.
[6] K. F. Babu, M. A. Kulandainathan, I. Katsounaros, L. Rassaei, A. D. Burrows, P. R. Raithby, and F. Marken, *Electrochem. Commun.* 2010, 12, 632.
[7] R. Tang, S. Zhou, L. Zhang, and L. Yin, *Adv. Funct. Mater.* 2018, 28, 1706154.
[8] K. J. Pyper, J. E. Yourey, and B. M. Bartlett, *J. Phys. Chem. C* 2013, 117, 24726.
[9] L. Xi, P. D. Tran, S. Y. Chiam, P. S. Bassi, W. F. Mak, H. K. Mulmudi, S. K. Batabyal, J. Barber, J. S. C. Loo, and L. H. Wong, *J. Phy. Chem. C* 2012, 116, 13884.
[10] J. Xiao, H. Huang, Q. Huang, X. Li, X. Hou, L. Zhao, R. Ma, H. Chen, and Y. Li, *Appl. Catal. B Environ.* 2017, 212, 89.
[11] J. Y. Kim, D. H. Youn, K. Kang, and J. S. Lee, *Angew. Chem. Int. Ed.* 2016, 55, 1.
[12] G. Liu, Y. Zhao, K. Wang, D. He, R. Yao, and J. Li, *ACS Sustainable Chem. Eng.* 2018, 6, 2353.
[13] Q. Rui, L. Wang, Y. Zhang, C. Feng, B. Zhang, S. Fu, H. Guo, H. Hu, and Y. Bi, *J. Mater. Chem. A* 2018, 6, 7021.
[14] F. Malara, A. Minguzzi, M. Marelli, S. Morandi, R. Psaro, V. D. Santo, and A. Naldoni, *ACS Catal.* 2015, 5, 5292.
[15] P. Zhang, T. Wang, X. Chang, L. Zhang, and J. Gong, *Angew.Chem. Int. Ed.* 2016, 55, 5851.
[16] Y.-J. Dong, J.-F. Liao, Z.-C. Kong, Y.-F. Xu, Z.-J. Chen, H.-Y. Chen, D.-B. Kuang, D. Fenske, and C.-Y. Su, *Appl. Catal. B Environ.* 2018, 237, 9.
